# Seasonal biological carryover dominates northern vegetation growth

**DOI:** 10.1038/s41467-021-21223-2

**Published:** 2021-02-12

**Authors:** Xu Lian, Shilong Piao, Anping Chen, Kai Wang, Xiangyi Li, Wolfgang Buermann, Chris Huntingford, Josep Peñuelas, Hao Xu, Ranga B. Myneni

**Affiliations:** 1 Sino-French Institute for Earth System Science, College of Urban and Environmental Sciences, Peking University, Beijing, China; 2 Key Laboratory of Alpine Ecology, Institute of Tibetan Plateau Research, Chinese Academy of Sciences, Beijing, China; 3 Center for Excellence in Tibetan Earth Science, Chinese Academy of Sciences, Beijing, China; 4 Department of Biology and Graduate Degree Program in Ecology, Colorado State University, Fort Collins, CO USA; 5 Institute of Geography, Augsburg University, Augsburg, Germany; 6 Institute of the Environment and Sustainability, University of California, Los Angeles, Los Angeles, CA USA; 7 UK Centre for Ecology and Hydrology, Wallingford, Oxfordshire UK; 8 CREAF, Cerdanyola del Valles, Barcelona, Catalonia Spain; 9 CSIC, Global Ecology Unit CREAF-CSIC-UAB, Bellaterra, Barcelona, Catalonia Spain; 10 Department of Earth and Environment, Boston University, Boston, MA USA

**Keywords:** Carbon cycle, Climate-change ecology

## Abstract

The state of ecosystems is influenced strongly by their past, and describing this carryover effect is important to accurately forecast their future behaviors. However, the strength and persistence of this carryover effect on ecosystem dynamics in comparison to that of simultaneous environmental drivers are still poorly understood. Here, we show that vegetation growth carryover (VGC), defined as the effect of present states of vegetation on subsequent growth, exerts strong positive impacts on seasonal vegetation growth over the Northern Hemisphere. In particular, this VGC of early growing-season vegetation growth is even stronger than past and co-occurring climate on determining peak-to-late season vegetation growth, and is the primary contributor to the recently observed annual greening trend. The effect of seasonal VGC persists into the subsequent year but not further. Current process-based ecosystem models greatly underestimate the VGC effect, and may therefore underestimate the CO_2_ sequestration potential of northern vegetation under future warming.

## Abstract (web summary)

The future of terrestrial systems is influenced by their past, but this carryover effect is rarely quantified. Here, the authors provide the first quantitative evidence that a greener spring begets a greener summer and autumn, and that this carryover effect is even stronger than climate drivers.

## Introduction

Biological cycles include many successional growth periods in which the past and the present are tightly connected. In such temporally connected dynamical systems, transient carryover of the near past is commonly observed for many state variables^1–3^. The carryover effect of biological states has been extensively documented in biomedical research, for example, describing the phenomenon where the effects of medical treatments can carry over from one to another in repeated clinical experiments^4^. This biological carryover effect is also widely existent in plant science^2,4–6^. The life-cycle continuity of plant growth implies that present states of vegetation growth may intrinsically affect subsequent growths, which is a type of biological memory^2,7^, and can be referred to as vegetation-growth carryover (VGC). The VGC effect could potentially control the pattern of seasonal-to-interannual variations of vegetation growth. For example, a tree may maintain a greening signal by cumulatively enhancing carbon uptake^8^, resulting in extra storage of photosynthate and more substantial leaves and roots. Such structural change of plants may then boost their resistance to climate fluctuations^9^ and emerging disturbances^10^, unless increasing water and heat stress exceeds the tolerance of sustainable tree growth^11^. The critical question is thus how strong this VGC effect is, particularly when compared against concurrently changing environmental conditions that also influence the present state of vegetation growth.

Projections of future vegetation and carbon uptake changes, including ecosystem capacity to offset CO_2_ emissions, are highly uncertain^12,13^, primarily due to our limited understanding of the mechanisms that govern vegetation growth dynamics. Surprisingly, while the concept of vegetation carryover effect is not new, and some key analyses searching for evidence of the VGC effect do exist, they are often focused on the short-term carryover^14^ or limited in their spatial scope^15^. Over a broad geographical range, it remains unclear how substantial the role of the VGC effect is in contributing to current and future vegetation growth and carbon cycle, particularly in comparison to that of abiotic factors (such as immediate and lagged impacts of climate). Indeed, the regulation of vegetation growth by abiotic factors, particularly climate and associated episodic climate extremes, has been extensively investigated and fairly well understood^16–22^. It is generally accepted that climate variation is the primary driver of seasonal-to-interannual dynamics of vegetation growth and associated carbon uptake over the Northern Hemisphere (NH)^16–18^. Importantly, climate change in the early growing season (EGS) may substantially influence vegetation growth of late seasons through, for instance, modulating plant transpiration^19,23–26^ and snow melting^27,28^, both leading to changes in soil moisture that can propagate into late seasons^25^. This climatic legacy effect via complex vegetation–soil–climate interactions has been now included in many state-of-the-art terrestrial biosphere models^29^. Still, these models produce a wide divergence in the estimates of vegetation growth and carbon uptake^12,13^, suggesting that some related mechanisms are either missing or incorrectly parameterized.

The interseasonal connections of vegetation growth has received increasing attention in recent years. The legacy effect of EGS vegetation growth on mid-to-late-season growth through transpiration-regulated soil moisture changes^19,23,25,26^ can be categorized as an exogenous memory effect under the theoretical framework of ecological memory by Ogle et al.^2^. Different from the legacy effect of climate anomalies or the exogenous memory of vegetation growth, the VGC effect in this work emphasizes how the carryover of the early vegetation structure may contribute to vegetation growth of the following seasons, or an endogenous memory effect according to the framework of Ogle et al.^2^.

In this study, we hypothesize that the VGC has played a critical role in regulating the seasonal-to-interannual trajectory of vegetation growth. To test this hypothesis, we quantify the impact of VGC on NH vegetation growth with a large set of measurements, including satellite, eddy covariance (EC), and tree-ring chronologies, and compare the size of this effect against that of immediate and lagged impacts of climate change (see “Methods”). Our work provides quantitative evidence that peak-to-late season vegetation productivity and greenness are primarily determined by a successful start of the growing season (via the interseasonal VGC effect), rather than by a transient or lagged response to climate. This carryover of seasonal vegetation productivity also contributes to annual vegetation growth across consecutive years.

## Results and discussion

### Interseasonal VGC dominates peak-to-late-season growth

**Fig. 1:**
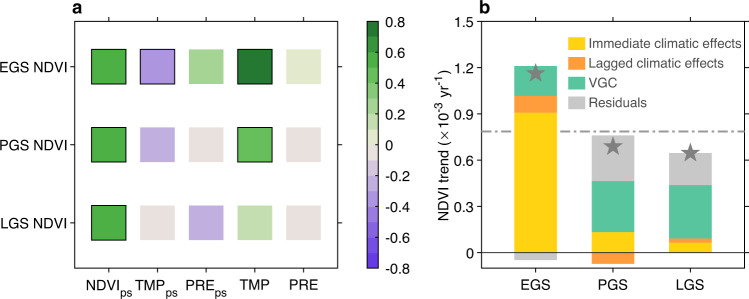
Satellite-based vegetation growth carryover versus climatic effects. **a** Partial correlation coefficients between 35-year seasonal Normalized Difference Vegetation Index (NDVI) time series and concurrent climatic factors (temperature, TMP and precipitation, PRE), and climatic factors (TMP_ps_ and PRE_ps_) and NDVI (NDVI_ps_) of the preceding season. The subscript ps denotes values for the immediately preceding season, except that ps for early growing-season (EGS) NDVI refers to NDVI of the preceding late growing season (LGS). Squares with black outline show statistically significant correlations at the 95% confidence level. **b** Individual contributions of the vegetation growth carryover (VGC) effect and the immediate and lagged climatic effects to seasonal NDVI trends over the 35 years (1982–2016) (see “Methods”). The gray dashed line indicates the observed trend of the growing-season mean NDVI over the Northern Hemisphere, and the gray stars indicate the observed NDVI trend of each season.

We first examined the VGC effect at the seasonal scale, using satellite-derived Normalized Difference Vegetation Index (NDVI, see “Methods”) for the 1982–2016 period. We defined the dormancy season (DS) and three periods of the growing season, i.e., EGS, peak growing season (PGS), and late growing season (LGS), based on phenological metrics (see “Methods”). The partial autocorrelation calculated for NDVI time series of two consecutive seasons, after factoring out concurrent and preceding climatic impacts, provides an estimate of the interseasonal VGC effects (see “Methods”). At the hemispheric scale, our analyses show a significant (*p* < 0.05) control of the NDVI of the preceding season (NDVIps) on the interannual variations of seasonal NDVI for all three active growing seasons (Fig. 1a). This VGC effect is also consistently positive across the majority (79%, 89%, and 94% for EGS, PGS, and LGS, respectively) of northern vegetated areas (Supplementary Fig. 1). Although the EGS NDVI is more strongly correlated with EGS temperature, the PGS and LGS NDVIs are most strongly correlated with NDVIps, rather than climate drivers (Fig. 1a). This VGC is particularly important for understanding vegetation growth in LGS, when climate is known to have a weak explanatory power^30,31^.

The primary role of NDVIps in driving subsequent PGS and LGS NDVI variations is reaffirmed by conducting partial correlations with detrended anomalies of all variables (Supplementary Fig. 2), implying a robust coupling of seasonal vegetation growth within a specific calendar year. Furthermore, the robustness of the satellite-identified positive VGC effect dominating vegetation growth in PGS and LGS is also verified by examining other satellite-derived vegetation growth proxies, including leaf area index (LAI) and gross primary productivity (GPP) (see “Methods”; results in Supplementary Fig. 3). Meanwhile, we also noticed some difference between GPP- and NDVI-derived LGS-to-EGS VGC effect in some mid-to-high northern latitudes (Supplementary Fig. 4d). Negative LGS-to-EGS VGC effect has been found over eastern U.S., North China, and western and central Russia based on the GPP dataset (Supplementary Fig. 4d), suggesting greater uncertainties in LGS-to-EGS VGC effect than EGS-to-PGS and PGS-to-LGS VGC effects.

In contrast to the consistently strong and positive VGC impact, the strength and direction of climatic factors in determining the interannual variation of seasonal NDVI, including their immediate and lagged impacts^16–20^, is highly variable between seasons and across regions (Fig. 1a and Supplementary Figs. 5 and 6). At the hemispheric scale, concurrent seasonal temperature is a primary and positive factor controlling the interannual variation of EGS NDVI during the last 35 years (partial correlation, *r*_*p*_ = 0.83, *p* < 0.01). The dominance of EGS temperature on EGS vegetation growth is also consistently observed when analyzing other satellite-based vegetation proxies of LAI and GPP (Supplementary Fig. 4). However, temperature has a much weaker impact during PGS (*r*_*p*_ = 0.42, *p* < 0.05) and LGS (*r*_*p*_ = 0.12, *p* > 0.05) (Fig. 1a). Although higher concurrent temperature generally stimulates vegetation activity in EGS across most of the northern vegetated areas (Supplementary Fig. 5a), it has emerged as a limit to PGS and LGS vegetation growth for most of the warm mid-latitudes and some of the high latitudes (Supplementary Fig. 5b, c). Additionally, temperature also has a negative legacy effect on vegetation growth in the subsequent season (Fig. 1a), most significantly for the DS-to-EGS legacy effect (*r*_*p*_ = −0.42, *p* < 0.05). This adverse legacy effect of DS temperature is likely due to the lower chilling accumulation required for leaf unfolding in EGS caused by DS warming^32^. For precipitation, we find very weak and statistically insignificant immediate and lagged impacts on vegetation growth for all the seasons at the hemispheric scale (Fig. 1a). This weak precipitation impact is likely due to a spatial cancelling-out of the positive effects at the water-limited mid-latitudes by the negative effects at high latitudes (more precipitation is often concurrent with increased cloudiness and reduced solar radiation reaching vegetation canopies) (Supplementary Figs. 5d–f and 6d–f).

For each season, we further derived the individual contributions of VGC, as well as the immediate and lagged climatic effects, to the 35-year NDVI trends (see “Methods”). The effects of temperature and precipitation were here combined as a single variable of climatic effects. As expected, the strong observed EGS greening trend (0.0012 yr^−1^, *p* < 0.05) is predominately attributed to the concurrent climate change (77%), particularly EGS warming that stimulates earlier phenology, followed by smaller but non-negligible contributions from climate (8%) in the preceding DS and vegetation growth in the preceding LGS (17%) (Fig. 1b). However, for PGS and LGS, about half of the observed greening trends (48% and 54%, respectively) are attributed to greening in the preceding season, supporting the notion of a strong positive biological carryover between seasons. In comparison, climate, including its immediate and lagged effects, plays a much smaller role in PGS and LGS greening (EGS climate may even cause a negative lagged impact on the PGS greening trend; Fig. 1b). Hence, warming-induced greening in EGS persists into the mid-to-late growing season, and has been the primary source for the overall satellite-observed NH growing-season greening over the last few decades^33,34^. It is interesting to note that PGS is the season whose interannual productivity variations most strongly correlate with that of the growing-season mean^35^, and trend of vegetation growth most similar to that of the growing-season mean (Fig. 1b). However, our results demonstrate that the higher peak growth rate in PGS is primarily inherited from greening of the preceding EGS (48%) rather than from direct contributions of PGS climate change (20%) (Fig. 1b).

Considering the substantial fraction of unexplained variance of observed vegetation growth in PGS and LGS after accounting for the climate and VGC effect of the concurrent and immediate precedent seasons (residuals in Fig. 1b), we further investigated the residual changes of PGS and LGS NDVI with vegetation and climatic factors in the previous year (see “Methods”). At the hemispheric scale, about 58% of the residuals of PGS NDVI changes can be explained by all the factors collectively (Supplementary Fig. 7c). NDVI of the previous LGS significantly correlates to PGS NDVI residuals (*r*_*p*_ = 0.55, *p* < 0.05), and contributes the most to the variance of residuals (Supplementary Fig. 7a). Among the climatic factors, PGS precipitation of the previous year shows the strongest correlation (*r*_*p*_ = 0.31, *p* = 0.09) with PGS NDVI residuals (Supplementary Fig. S7a), indicating a strong legacy effect of precipitation anomalies (such as droughts) on PGS vegetation growth. None of the considered factors shows a significant (*p* > 0.05) correlation with LGS NDVI residuals, and collectively they explain about 20% of LGS NDVI variance (Supplementary Fig. S7c).

**Fig. 2:**
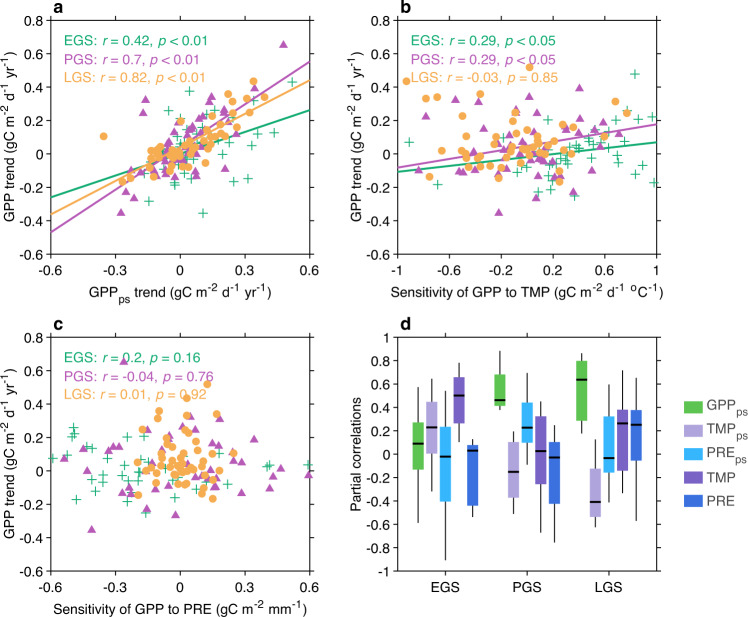
Site-based vegetation growth carryover versus climatic effects. **a** Scatterplot of the gross primary productivity (GPP) trend for each season against that of the preceding season across 50 FLUXNET sites. **b** Scatterplot of the GPP trend for each season against the GPP sensitivity to concurrent temperature across 50 FLUXNET sites. **c** Scatterplot of the GPP trend for each season against the GPP sensitivity to concurrent precipitation across 50 FLUXNET sites. In all panels, best-fitting straight lines are shown for significant relationships, along with related statistics as annotated. **d** Partial correlation coefficients between anomalies of seasonal GPP changes and that of each driving factor, based on measurements from 11 Ameriflux sites. Boxplots show the maximum, upper-quartile, median, lower-quartile, and minimum of the distribution across sites. EGS, PGS, and LGS represent early, peak, and late growing season, respectively.

We further examined the VGC effect and vegetation–climate connections with seasonal GPP data from the global FLUXNET EC network. The short temporal coverage of EC records prevents calculating temporal correlations, we hence analyzed the relationship between the trend of GPP and that of its potential drivers across 50 available flux-tower sites (“Methods”). Consistent with the satellite-based findings, we discovered strong positive cross-site correlations between the trend of GPP and that of its preceding values for all the growing seasons (Pearson correlation, *r* = 0.42, 0.70, and 0.82 for EGS, PGS, and LGS, respectively, *p* < 0.01 in all cases; Fig. 2a). However, temperature and precipitation changes cannot account for the cross-site variation of GPP trends for any season (Supplementary Fig. 8), even though EGS temperature is identified as the primary driver of satellite-based EGS NDVI changes (Fig. 1a). The weak cross-site correlation with climatic variables may be overshadowed by the biome-dependent sensitivity of GPP to climate^16,17^. To test this, we examined the cross-site relationship between the GPP trend and its climatic sensitivities (“Methods”). We found a significant positive correlation between the EGS GPP trend and its sensitivity to temperature (*r* = 0.29, *p* < 0.05) (Fig. 2b), supporting that EGS warming controls EGS greening patterns. For all the other cases, the insignificant (*p* > 0.05) relationship between the GPP trend and its climatic sensitivity (Fig. 2b, c) supports a weak climatic impact.

In addition to these global datasets, we also used long-term GPP measurements from 11 AmeriFlux EC sites (“Methods”) to characterize temporal relationships between vegetation growth and climate. Results of this analysis again confirm our main findings: EGS temperature is the primary determinant of EGS GPP (cross-site median correlation: *r*_*p*_ = 0.52, *p* < 0.05), which is then carried over to dominate the variance of GPP in PGS (*r*_*p*_ = 0.43, *p* < 0.05) and LGS (*r*_*p*_ = 0.62, *p* < 0.05) (Fig. 2d). GPP of PGS and LGS also show varied signs of correlation with other climate factors in the previous year (Supplementary Fig. 7), which collectively explain 30–72% and 16–81% of the GPP residual variance, respectively.

**Fig. 3:**
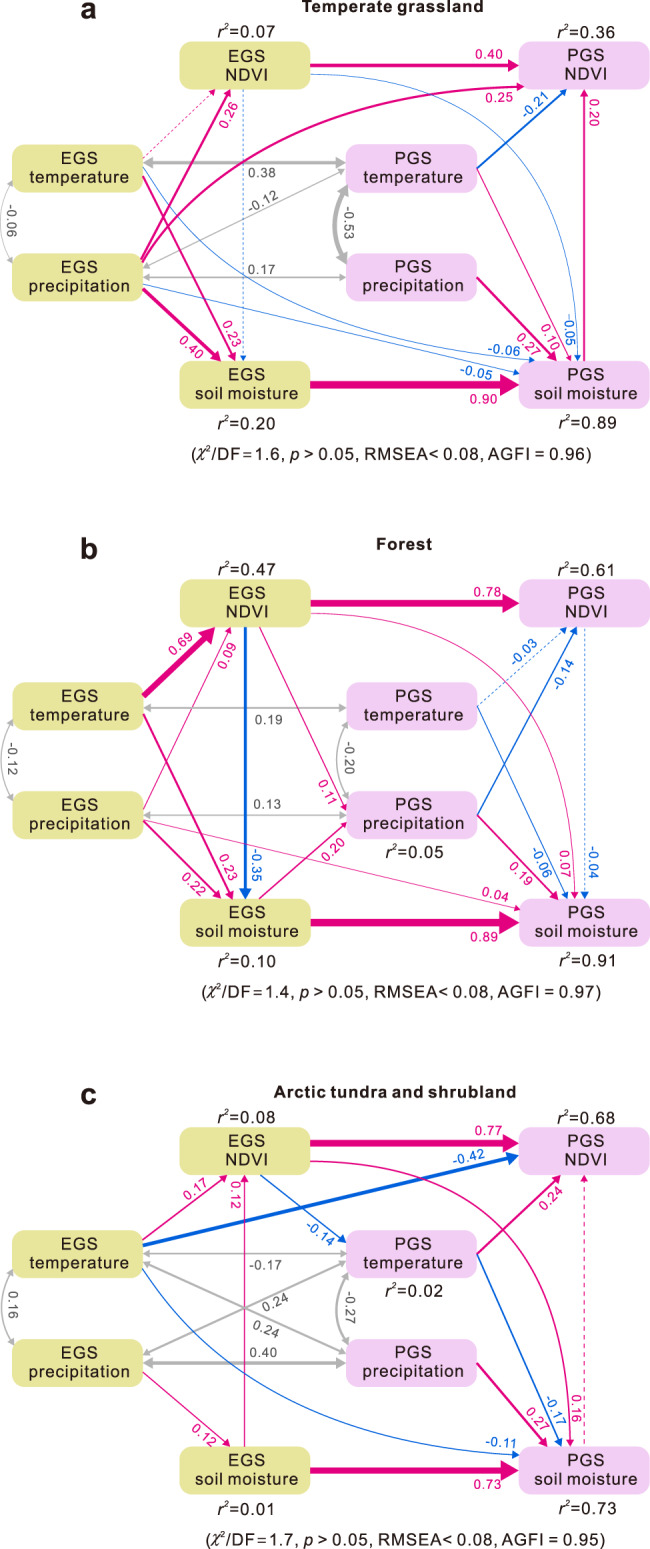
Pathways for early-season factors controlling peak-season growth. Structural equation modeling (SEM) analyses were conducted for three main vegetation types: temperate grassland (Tibetan Plateau excluded) (**a**), forest (**b**), and arctic tundra and shrubland (**c**) (see Supplementary Fig. 9). Double-headed gray arrows indicate covariance between variables. Single-headed arrows indicate the hypothesized direction of causation, with positive and negative relationships in pink and blue, respectively. Solid lines represent relationships that are significant statistically (*p* < 0.05), and hatched lines represent relationships that are not significant statistically (*p* > 0.05). Arrow thickness is proportional to the strength of the relationship and to the standard path coefficients adjacent to each arrow. The explained variance (*r*^2^) is labeled alongside each response variable in the model. EGS and PGS represent early and peak growing season, respectively.

Our observed interseasonal connection in vegetation activity may also be modulated by indirect non-biological pathways, for example, soil moisture anomalies caused by vegetation changes persisting into the next season^19,22,25,36^. These different mechanisms imply potentially multiple simultaneous pathways for the interseasonal interactions between vegetation, climate, and soil moisture status. To quantify the complex pathways underpinning interseasonal vegetation–climate–soil interactions, we constructed structural equation models (SEMs), forced with satellite-based NDVI and soil moisture, and climatic variables (see “Methods”). We allowed for broad biome differences by grouping northern vegetation into three main vegetation types of temperate grassland, forest, and arctic tundra and shrubland, based on satellite-derived land-cover maps (“Methods”; Supplementary Fig. 9). Figure 3 shows all pathways of the EGS–PGS connection (for other interseasonal linkages see Supplementary Fig. 10). The SEM analysis identifies the significant positive influence of EGS vegetation growth on that of PGS, explaining the largest fraction of PGS NDVI variations for all vegetation types (Fig. 3). This result provides further support for strong VGC between EGS and PGS vegetation growth. This EGS-to-PGS VGC effect is robust by further demonstrating that for all vegetation types, years with greener EGSs (under favorable climates) generally have greener PGSs, and accordingly, years with browner EGSs (under unfavorable climates) tend to have browner PGSs (Supplementary Fig. 11).

In parallel to the interseasonal vegetation growth carryover, we also diagnosed a strong interseasonal carryover effect of soil moisture, where local soil moisture status in PGS links tightly to that in EGS (Fig. 3). However, the indirect impact of EGS vegetation growth on PGS vegetation via this soil moisture pathway may be weaker than previously thought^22^. For grassland where water is often the dominant limiting factor, PGS soil moisture does significantly influence PGS productivity, yet the amount of soil moisture in EGS is controlled predominantly by EGS climate rather than vegetation (Fig. 3a). For forest-dominated ecosystems, EGS greening does significantly dry out the soil, causing a soil moisture deficit that is further carried over to the PGS^25^. However, this moisture deficit has limited impacts on restraining PGS forest growth (Fig. 3b), likely due to the deep root system that can access water reservoirs in deep soil layers^37^. For arctic tundra and shrubland, temperature is a key limiting factor^38^, and thus the vegetation–soil moisture interaction is relatively weak for both EGS and PGS seasons (Fig. 3c). Furthermore, we also find that the VGC effect is more dominant than soil moisture carryover effect for both EGS (from DS or the preceding LGS; Supplementary Fig. 10a, c, e) and LGS NDVI (from PGS; Supplementary Fig. 10b, d, f).

### The persistence of the VGC effect into the subsequent year

**Fig. 4:**
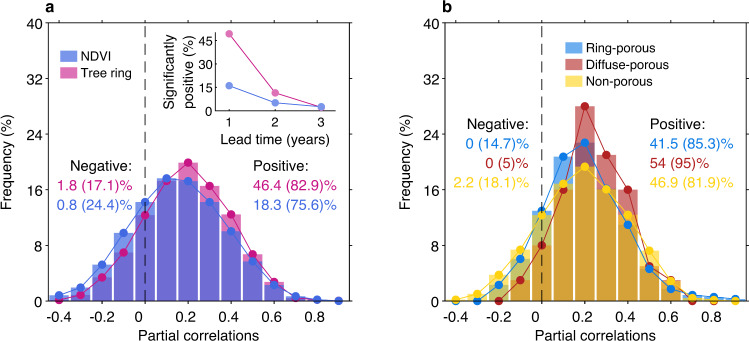
Observed interannual vegetation growth carryover. **a** The histogram shows the frequency distribution of the partial correlations between Normalized Difference Vegetation Index (NDVI), or tree-ring width, of each year and that of the preceding year, after controlling for the climatic variable of both years. **b** Frequency distribution of the partial correlations between tree ring width of each year and that of the preceding year, for ring-porous, diffuse-porous, and non-porous trees (classification details in Supplementary Table 1). Numbers show the percentage of grids (or tree samples) showing statistically significant (*p* < 0.05) negative/positive partial correlations, with the bracketed numbers showing the percentage of all negative/positive correlations. The inset plot of **a** shows the percentage of grids (or tree samples) showing significantly (*p* < 0.05) positive correlation between present and preceding years’ NDVI (or tree ring width) with lead times ranging from 1 to 3 years. Note that all variables are detrended before performing partial correlation analysis.

In order to examine whether this VGC effect operates at longer time scales of multiple years, we next performed lagged partial autocorrelations with interannual anomalies of satellite-observed NDVI and 2739 standardized tree-ring width (TRW) records (see “Methods”). For a time lag of 1 year, a positive interannual VGC is present across northern lands, with 75.6% of vegetated areas (for NDVI) and 82.9% of the tree-ring samples (for TRW) showing positive lagged correlations (Fig. 4a and Supplementary Fig. 12). This positive interannual VGC indicates that a greener year is often followed by another greener year. The positive VGC is statistically significant (*p* < 0.05) for 18.3% of northern areas based on NDVI, but noting it is significant for 46.4% of the tree-ring samples that cover much longer periods (Fig. 4a). The positive interannual VGC effect is most significant at high latitudes, particularly over northern North America and East Siberia (Supplementary Fig. 12). Interestingly, by further grouping tree species into ring-porous, diffuse-porous and non-porous species (Supplementary Table 1), we found stronger interannual VGC effect for diffuse-porous species (95.0% positive) than for ring-porous (85.3% positive) and non-porous species (81.9% positive) (Fig. 4b). This observation suggests substantial influence of wood phenology on the strength of vegetation growth carryover, and diffuse-porous species whose woody growth is more concentrated in later growing season are more likely to carry transient growth anomalies over to the subsequent year. By contrast, only a few locations (including central Siberia, eastern Europe, and some semi-arid regions) have a negative yet generally insignificant (*p* > 0.05) interannual VGC (Supplementary Fig. 12). If the time lags are extended to 2 years, the positive correlation between current-year NDVI (or TRW) and that of 2 years earlier is significant for only 14% of tree-ring samples or 5% of the total vegetated area (for NDVI). If time lags of 3 years are considered, the lagged correlation is found to be close to zero (Fig. 4a). Previous studies have reported stronger legacies of severe drought episodes (e.g., >2 SD from the mean climatic water deficit) lasting 2–4 years^20,21^. However, for interannual anomalies (=1 SD) of vegetation growth that is much less deviated from the multi-year average than severe drought anomalies, the VGC effect can be carried over to the next year but rarely to years after that.

### Terrestrial biosphere models underestimate the VGC effect

**Fig. 5:**
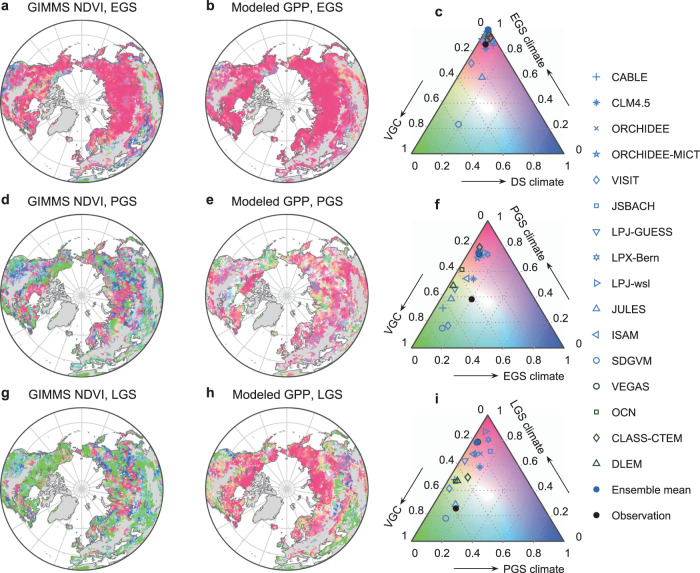
Observed versus modeled vegetation growth carryover effects. Spatial patterns show the relative contributions of vegetation growth carryover (VGC) and climatic factors of the present and preceding seasons to the interannual variations of Normalized Difference Vegetation Index (NDVI) or gross primary productivity (GPP). Maps in the left column represent satellite-observed NDVI, and those in the right are the model ensemble-mean GPP for early growing-season (EGS) (**a**, **b**), peak growing-season (PGS) (**d**, **e**), and late growing-season (LGS) (**g**, **h**). Ternary diagrams in **c**, **f**, **i** show the relative fraction of global vegetated areas where the interannual trend of GPP (or NDVI) is dominated by each different driver (corresponding to the maps in each row). Both percentages of the model ensemble-mean (closed blue circles) and the individual models (open symbols) and of satellite observation-based estimates (closed black circles) are shown.

Process-based terrestrial biosphere models are a useful tool for predicting vegetation growth and examining the associated complex mechanisms^29,31^. We next assessed 16 terrestrial biosphere models participating in the TRENDY intercomparison project (“Methods” and Supplementary Table 2) for their ability to capture the dominant factors contributing to the satellite-observed greenness changes in each season. By comparing modeled GPP (Fig. 5b, of multi-model mean) against satellite-observed greenness (Fig. 5a), we found that the models correctly identified EGS temperature as the primary factor controlling interannual variations of EGS vegetation activity for most northern areas. In 15 out of the 16 models, areas where EGS temperature is the primary driver of concurrent GPP variations were found to have the largest spatial coverage among all potential driving factors (Fig. 5c). However, the satellite-identified dominance of VGC effects in PGS and LGS vegetation growth for much of the northern lands (42% for PGS and 58% for LGS; Fig. 5d, g and Supplementary Fig. 13b, c) is significantly underestimated by models (19% for both PGS and LGS; Fig. 5e, h and Supplementary Fig. 13e, f). Multi-model averaged results indicate an overwhelming fraction of vegetated land is instead dominated by the immediate climatic effects for both PGS (75%) and LGS (78%). Specifically, 10 out of the 16 models significantly underestimate the proportion of VGC-dominated areas for PGS vegetation greening, and nearly all (15) of the models significantly underestimate the proportion for LGS vegetation growth, despite the strong intermodel discrepancy in the proportion of projected VGC-dominated areas (from 14% in LPX-Bern to 72% in SDGVM for PGS and from 9% in LPJ-wsl to 75% in SDGVM for LGS) (Fig. 5f, i).

With rising atmospheric CO_2_ concentrations and anticipated warmer climate, Earth system models that simulate stronger VGC effects tend to project higher carbon uptake potentials over northern ecosystems (Supplementary Fig. 14). To improve estimates of how the global carbon cycle will evolve in the decades ahead, it is critical to diagnose the causes of this underrepresentation of modeled VGC effects. We therefore compared the three models that best identify the areas identified by satellite where VGC dominates vegetation growth versus the three models that least capture it (based on Fig. 5f, i). As expected, we find that the models with the best representation of the VGC effect produce better estimates of PGS and LGS levels of greenness based on EGS growth levels, for all the three major biomes (Supplementary Fig. 15a, c, e). Conversely, for the models that fail to replicate the VGC effect, modeled years with the greenest EGSs do not necessarily imply a greener PGS or LGS, especially for temperate grasslands and forests (Supplementary Fig. 15b, d).

**Fig. 6:**
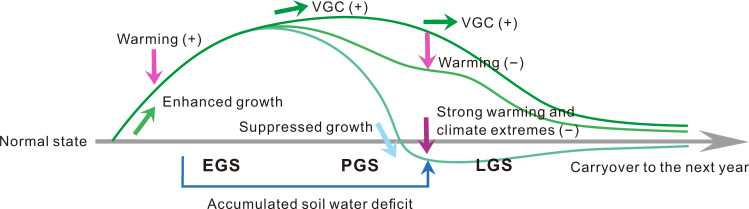
Schematic representation of the vegetation growth carryover. The green curves indicate anomalies of vegetation greenness/productivity in response to climatic changes and disturbances, relative to the climatological seasonal cycle (the gray line). Early growing-season (EGS) warming stimulates extra vegetation growth and productivity, and this ecological benefit can persist into peak and late growing-season (PGS and LGS) and even the subsequent year because vegetation growth is largely determined by its prior states (i.e., the vegetation growth carryover, VGC). This greening signal from EGS, however, may be suppressed or even reversed for some locations due to climate extremes or soil moisture deficit legacy from EGS. The symbols − and + in each bracket represent either a negative or positive force respectively imposed on terrestrial vegetation.

Guided by the identified drivers from our empirical analyses (Figs. 1–3), we tested the hypothesis that the interseasonal inconsistency in modeled greening trends is related to sensitivity biases of vegetation productivity responses to climate variation (Fig. 6). Comparison of satellite-based and modeled sensitivities of PGS and LGS vegetation productivity to climatic variables confirms this hypothesis. For both PGS and LGS, models with better VGC representation show very similar spatial patterns of productivity sensitivities to temperature and precipitation as that derived from observations (Supplementary Figs. 16a, b, d, e and 18a, b, d, e). However, models underestimating the VGC effect broadly overestimate the climate sensitivity. In specific, for PGS, models underestimating the VGC effect severely overestimate the magnitude and extent of the negative impact of PGS warming and precipitation decrease on vegetation productivity in temperate regions (Supplementary Fig. 16c) and some semi-arid areas (Supplementary Figs. 16f and 17b), respectively. Similarly, for LGS, models that underestimate the VGC effect tend to overestimate the positive effects of LGS temperature on vegetation productivity in cold areas (>45°N) (Supplementary Fig. S18a, c, d, f).

### Conclusions and implications

In summary, our analyses reveal strong biological carryover effects of vegetation growth and productivity across succeeding seasons and years, providing new insights into how vegetation changes under global warming. The VGC effect represents a key yet often underappreciated pathway through which warmer EGSs and associated earlier plant phenology subsequently enhance plant productivity in the mid-to-late growing season, which can further persist into the following year (Fig. 6). Without considering this biological carryover of vegetation growth, some previous studies suggest an overly negative impacts of EGS warming on PGS/LGS vegetation growth, in particular, through triggering earlier transpiration and associated soil moisture deficits^19,23,36^. Yet, despite the potential for raised soil moisture deficits, we find the strong VGC effects can override this negative abiotic legacy impacts, with greener EGSs ensuring lush PGS vegetation (Fig. 6). Hence, warming in EGS not only augments concurrent vegetation growth and carbon uptake but also has a positive legacy impact on the following PGS and LGS vegetation carbon sequestration, ultimately enhancing the annual carbon sink. However, it is important to bear in mind that, while the beneficial VGC effect of EGS vegetation growth can override immediate and time-lagged climatic impact under the present climate, whether this stronger VGC effect will continue with future warmer climate remains an open question (Fig. 6). Processes involved in the lagged vegetation responses to precedent climate, soil, and growth conditions are highly complex and often non-linear^6,39,40^. For example, summer climate extremes, which are often associated with large-scale climate oscillations and partly contributed by enhanced EGS vegetation growth^25^, could trigger severe tree mortality and fires that nullify any positive carryover effect from EGS (Fig. 6). Recent advances in statistical modelling and machine learning^6,39,41^ may provide useful tools for a better understanding of such non-linear vegetation responses.

We also find poor representation of the VGC effect in dynamic vegetation models, and as this likely influences predictive capacity of future global carbon cycle changes, a major research challenge is to better simulate biological processes related to this carryover effect. Tackling this challenge requires not only using satellite and ground measurements to refine existing parameterizations, but also using leaf-level measurements to understand the physiological mechanisms controlling VGC patterns and to incorporate new process representation in model components. Long-term manipulative field experiments will also be useful to better characterize VGC features under different imposed meteorological regimes and to provide key process parameters for future model improvements.

## Methods

### Satellite-based vegetation growth and land-cover maps

Normalized Difference Vegetation Index (NDVI) is commonly used as a proxy for vegetation greenness and photosynthetic activity. Here, NDVI data were obtained from the Global Inventory Monitoring and Modeling Studies (GIMMS) third-generation NDVI product (NDVI3g) based on retrievals from sensors on the Advanced Very High Resolution Radiometer (AVHRR)^42,43^. The GIMMS NDVI3g dataset is available at a spatial resolution of 8 × 8 km^2^ and a biweekly temporal resolution, covering the 1982–2016 period. We composited the biweekly NDVI to monthly values by selecting the highest values.

Considering that NDVI may saturate in densely vegetated areas, we also included two other satellite-based products, LAI and GPP, to independently verify the robustness of NDVI-based findings. Biweekly maps of the global land LAI were derived from the GIMMS AVHRR LAI3g^44^, with a spatial resolution of 8 × 8 km^2^ for the period 1982–2016. The monthly gridded GPP maps at 0.5° spatial resolution for 2001–2015 were derived from the remote sensing-based (RS) product of the FLUXCOM database^45,46^. This dataset was generated with upscaling approaches based on three machine learning algorithms that integrated EC-based carbon fluxes and satellite measurements from Moderate Resolution Imaging Spectroradiometer (MODIS)^45,46^.

The effects of climate and VGC on vegetation changes can vary among ecosystem types. Therefore, we investigated the climatic and VGC impacts separately for three major vegetation types of temperate grassland, forest (temperate and boreal), and arctic tundra and shrubland. We used the 300-m resolution global land-cover maps for 1992–2016 provided by the European Space Agency’s Climate Change Initiative (ESA-CCI)^47^ to delineate the three major vegetation types. These maps characterize the global surface using 37 land-cover classes defined by the United Nations Land Cover Classification System (UNLCCS). We grouped the original UNLCCS classes into forest, shrub, and grassland based on the cross-walking table provided by the ESA-CCI land cover product^47^. We did not consider shrub as a separate group in temperate regions, but assigned this type evenly to grasses and forests. In this study, temperate grassland was defined as water-limited grassland distributed in warm mid-latitude regions, but excluding temperature-limited grassland in pan-Arctic regions and the Tibetan Plateau. The forest type includes evergreen needleleaf forests, evergreen broadleaf forests, deciduous needleleaf forests, deciduous broadleaf forests, and mixed forests. The arctic tundra and shrubland was defined as temperature-limited grassland and shrubland over high latitudes (>50°N). We aggregated the land-cover maps into 0.5° resolution, and calculated the fraction of the above three vegetation types in each 0.5° grid. We only selected grid cells for which the dominant vegetation type occupied >60% of the grid area over the entire period of 1992–2016 (Supplementary Fig. 9) to minimize potential confounding impacts of other vegetation types and land cover conversions. We also masked northern ecosystems dominated by cropland, as for these locations, the seasonal vegetation growth is primarily controlled by human management practices such as irrigation, fertilization, cropping schedule, and multi-cropping, rather than environmental drivers.

### Climatic and soil moisture data

Environmental variables used here include temperature, precipitation, and soil moisture. Monthly time series of temperature and precipitation were obtained from the Climatic Research Unit (CRU) v4.0.1 dataset^48^. This gridded dataset, with a spatial resolution of 0.5°, was constructed by interpolation from meteorological stations based on spatial autocorrelation functions^48^. This climatic product also provides climatic forcing for TRENDY model simulations, ensuring better comparability between observed and modeled responses of ecosystems to climate. Daily root-zone soil moisture estimates with a spatial resolution of 0.25° over 1988–2016 were derived from the Global Land Evaporation Amsterdam Model (GLEAM) v3.2a^49^. The GLEAM data have fully assimilated microwave observations of precipitation, surface-soil moisture, and vegetation optical depth (VOD)^49^. GLEAM incorporates VOD as this enables estimates of the effects of water and heat stress and plant phenological changes on evapotranspiration^49^. This knowledge of vegetation response in turn allows characterisation of interactions between soil moisture and vegetation growth.

### EC measurements

We enhanced the reliability of remotely sensed seasonal vegetation–climate interactions by additional analyses using monthly GPP estimates and climatic variables from the FLUXNET2015 and AmeriFlux EC measurements. EC-based GPP values used here were estimated from the direct measurement of net ecosystem CO_2_ exchange by flux towers, combined with knowledge of plant light-response curves^50^. This FLUXNET2015 database consists of 212 sites that encompass 13 major vegetation types defined according to the classification system of the International Geosphere Biosphere Programme (IGBP). Here, we selected sites that provided at least 7 years of records, and excluded those labeled as cropland or falling into MODIS-based regions dominated by cropland, leading to a total of 50 EC sites for analysis. Since launched in 1996, the AmeriFlux observation network provides half-hourly or hourly flux records that allow for temporal correlation analyses. We obtained a subset of 11 AmeriFlux sites (CA-TP4, US-Los, US-Me2, US-Ne1, US-Ne2, US-Ne3, US-PFa, US-Ton, US-Uaf, US-Var, US-WCr) that provide at least 15 years of data, including GPP flux and meteorological variables.

### TRM chronologies

We obtained 2739 standardized TRM chronologies from the International Tree-Ring Data Bank (ITRDB)^51^ V713 dating to August 2017. All selected tree-ring samples are located in the NH (>30°N), and cover at least 25 years during 1901–2016. Each chronology is an average annual time series of standardized ring width measurements from typically 10 to 30 trees of the same species. We derived the standardized TRM series by detrending the raw TRM measurements based on the “cubic smoothing spline” approach^52^. This standardization removes low-frequency signals of wood growth associated with increasing tree age and trunk diameter, while still preserving interannual and interdecadal variabilities^51^. Site-level standard chronologies were generated by averaging tree-level standardized tree ring indices with a bi-weight robust mean^53^.

### Process-based ecosystem model simulations

We used an ensemble of 16 process-based terrestrial biosphere models participating in the TRENDY (trends in net land–atmosphere carbon exchange) v6 project^29^ that provide GPP outputs for 1982–2016. These models were CABLE, CLM4.5, ISAM, JSBACH, JULES, LPJ-GUESS, LPJ-wsl, LPX-Bern, ORCHIDEE, ORCHIDEE-MICT, SDGVM, VISIT, VEGAS, OCN, CLASS-CTEM, and DLEM (details in Supplementary Table 2). All these models performed the same set of factorial simulations following a standard experimental protocol^29^. In particular, we use TRENDY simulation S2 that was forced by varying both atmospheric CO_2_ and climate. The historical climatic fields were from the CRU-NCEP V8 dataset, which is a merged product of monthly CRU observations and a 6-h NCEP reanalysis. Global atmospheric CO_2_ concentrations were from a combination of ice-core records and the NOAA monitoring observations.

### Quantifying climatic and VGC effects on vegetation growth

We used satellite-derived NDVI and concurrent climatic data to identify covariation between the interannual variation of vegetation growth at northern latitudes (>30°N) of active vegetation growing seasons (EGS, PGS, or LGS). We considered the NDVI of the preceding season, as well as climatic variables (temperature and precipitation) of both the focused season and the preceding season, as driving factors of seasonal NDVI variations. Here we defined preceding-season NDVI for EGS as NDVI of the preceding LGS rather than the preceding DS, since vegetation in DS is dormant and the detection of NDVI is hampered by the presence of snow cover (in cold regions).

Periods of different seasons were not defined for each grid cell directly by temperature thresholds^19^ or fixed months across regions (e.g., spring as March–May)^25^. Instead, we account for actual phenological seasonality using the climatological mean seasonal cycle of satellite-derived monthly NDVI values for 1982–2016. We derived the dates for the start of the growing season (SOS) and the end of the growing season (EOS) across the NH (>30°N) from the time when the rate of the daily NDVI change interpolated from the original biweekly NDVI time series was highest and lowest, respectively^54^. Our analysis is based on the 35-year average of SOS and EOS, since we focus on interseasonal connections of mean vegetation growth states (greenness or productivity) instead of the shift of phenological events. PGS was defined as the two consecutive months with maximum NDVI values but not earlier than April or later than October. Grid cells with maximum NDVI occurring before April or after October were not considered. For regions with a growing season length of 3 months or less, PGS was defined as the month with the maximum NDVI value. Accordingly, EGS was defined as the period from the month containing the SOS date to the beginning of PGS, and LGS was from the end of PGS to the month of EOS. The remaining months of a year were defined as the DS. Compared to seasonal descriptions based on temperature thresholds^19^ or fixed months^25^, these definitions of seasons utilizing the timing of phenological events are more suitable for analyzing linkages between different stages of vegetation growth in the active growing season. We aggregated monthly NDVI for the three vegetation active seasons and then resampled the NDVI values to 0.5° to match the spatial resolution of the climatic data. The above definitions of seasons were also used for gridded EC measurements of GPP and climatic variables. For site-level analyses, we derived SOS and EOS values based on the derivative of the time series of daily GPP (i.e., the maximal/minimum second derivative value as the SOS/EOS^55^) that were smoothed with spline curves.

We quantified the contributions of climatic drivers (present- and preceding-season for both temperature, TMP and precipitation, PRE) and VGC (preceding-season NDVI) to observed NDVI trend during 1982–2016. This quantification was achieved by decomposing the 35-year linear trend of NDVI ($\frac{{{\mathrm{d}}{\mathrm{NDVI}}}}{{{\mathrm{d}}t}}$) for each season into the additive contributions of five components:

1 $$\frac{{{\mathrm{d}}Y}}{{{\mathrm{d}}t}} = 	\; \frac{{\partial Y}}{{\partial Y_{{\mathrm{{ps}}}}}}\frac{{{\mathrm{d}}Y_{ps}}}{{{\mathrm{d}}t}} + \frac{{\partial Y}}{{\partial {\mathrm{TMP}}_{{\mathrm{ps}}}}}\frac{{{\mathrm{d}}{\mathrm{TMP}}_{{\mathrm{ps}}}}}{{{\mathrm{d}}t}} + \frac{{\partial Y}}{{\partial {\mathrm{PRE}}_{{\mathrm{ps}}}}}\frac{{{\mathrm{d}}{\mathrm{PRE}}_{{\mathrm{ps}}}}}{{{\mathrm{d}}t}} + \frac{{\partial Y}}{{\partial {\mathrm{TMP}}}}\frac{{{\mathrm{d}}{\mathrm{TMP}}}}{{{\mathrm{d}}t}} + \frac{{\partial Y}}{{\partial {\mathrm{PRE}}}}\frac{{{\mathrm{d}}{\mathrm{PRE}}}}{{{\mathrm{d}}t}}+\varepsilon \\ = 	\, {\Delta}Y^{Y_{{\mathrm{ps}}}} + {\Delta}Y^{{\mathrm{TMP}}_{{\mathrm{ps}}}} + {\Delta}Y^{{\mathrm{PRE}}_{{\mathrm{ps}}}} + {\Delta}Y^{{\mathrm{TMP}}} + {\Delta}Y^{{\mathrm{PRE}}}+\varepsilon = {\Delta}Y^{Y_{{\mathrm{ps}}}} + \!{\Delta}Y^{{\mathrm{CLM}}_{{\mathrm{ps}}}} + {\Delta}Y^{{\mathrm{CLM}}}+\varepsilon$$

where $\frac{{\partial Y}}{{\partial X}}$ represents the sensitivity of *Y* (NDVI) to an explanatory variable *X* (NDVI_ps_, TMP_ps_, PRE_ps_, TMP, or PRE). These sensitivities were estimated as the regression coefficients of a multiple linear regression performed with NDVI against all listed explanatory variables for 1982–2016. $\frac{{{\mathrm{d}}Y}}{{{\mathrm{d}}t}}$ (or $\frac{{{\mathrm{d}}X}}{{{\mathrm{d}}t}}$) represents the linear trend of *Y* (or *X*) during 1982–2016. For different seasons, this trend was calculated as the slope of the simple linear regression of mean *Y* (or *X*) values against the year. Here, The NDVI trend during 1982–2016 ($\frac{{{\mathrm{d}}Y}}{{{\mathrm{d}}t}}$) was decomposed into the contribution of each variable *X* (Δ*Y*^*X*^), which was represented as the product of the partial derivative against that variable *X* as $\frac{{\partial Y}}{{\partial X}}$ and the concurrent trend of *X* itself as $\frac{{{\mathrm{d}}X}}{{{\mathrm{d}}t}}$. Note that the contributions of temperature and precipitation were combined to provide the total contribution of climate change to the trend of NDVI, for both the preceding season (${\Delta}Y^{{\mathrm{CLM}}_{{\mathrm{ps}}}}$) and the present season (${\Delta}Y^{{\mathrm{CLM}}}$). *ε* represents the difference between the observed and predicted *Y*. The approach given by Eq. (1) was conducted for each grid cell, and the total areally-averaged contribution of each factor to the trend of NDVI over the NH was calculated by averaging the decomposed contribution of factors (Δ*Y*^*X*^) across all the northern vegetated nonagricultural areas. In addition to satellite-observed analyses, we also conducted similar analyses with TRENDY model simulated GPP. The analyses were similar to that of NDVI-based, except that GPP was used to represent vegetation growth as the dependent variable.

We also applied a partial correlation analysis to assess the relationship between seasonal time series of NDVI and each driving factor while statistically controlling for potential covarying effects of the remaining set of factors. This analysis was performed for NDVI values averaged over the entire NH (30–90°N) (Fig. 1) and that for each pixel (Supplementary Figs. 1 and 4–6).

At the annual time scale, we again calculated partial correlations between NDVI of each year and that of the preceding year (and similarly for TRW), while statistically factoring out the covarying effects of climatic factors (temperature and precipitation of the present and preceding year). For comparability with standardized tree ring data, linear trends of all yearly time series were removed. Additionally, we quantified the persistence of the interannual VGC by calculating partial autocorrelation coefficients^20^ of NDVI and TRW time series, but with the lead time ranging from 1 to 3 years. For example, for a lead time *j* (years), we calculated the partial correlation between *Y*_*t*_ (NDVI or TRW, *t* is the present time) and *Y*_*t*−*j*_, while factoring out the covarying effects of all smaller lead periods (0, 1, 2, …, *j*−1).

As a further check of the detected NDVI-based seasonal ecosystem responses to different drivers, we also plotted the trend of GPP against that of climatic factors preceding-season GPP across all FLUXNET EC sites (Fig. 3). The majority of these EC sites are distributed in temperate climates with relatively homogeneous trends of temperature. Thus these sites alone do not encompass sufficient spatial variations of GPP response to warming. However, the sensitivity of GPP to temperature varies across sites of different ecosystem types. We therefore plotted the trend of GPP against the calculated temporal sensitivity of GPP to temperature across these EC sites, to understand such ecosystem dependence. For the 11 Ameriflux EC sites with sufficient temporal coverage, we quantified the partial correlations between time series of GPP and that of its driving factors, in the same manner as that conducted for satellite-observed NDVI.

In Eq. (1), residual *ε* represents the contributions of other drivers that could influence vegetation growth but were not included in this regression. The above analyses focus on the influence of factors in the concurrent and the immediately previous season on seasonal vegetation growth. An additional analysis was also performed to extend the time period to the entire previous year except the immediately neighboring season, by correlating the residual series with the same factors of the previous seasons. For example, for *ε* of PGS NDVI, we calculated its correlation with NDVI, TMP, and PRE of the previous PGS and LGS, and TMP and PRE of the previous DS (Supplementary Fig. 7).

We also used SEM to assess the direct and indirect pathways of how climatic factors and VGC influence vegetation changes across seasons for the three major vegetation types. SEM is a multivariate statistical approach that synthesizes path, factor, and maximum-likelihood analyses, and provides strong pointers to underlying deterministic processes. Compared with traditional multivariate analyses, SEM allows partitioning the direct and indirect effects that one variable may have on another, and is thus useful for exploring complex influence networks in ecosystems. In this model, the causality between soil moisture and vegetation greenness is determined by the sign of their correlation. Positive correlations indicate a dominance of soil moisture impact on vegetation (soil moisture stimulates growth), while negative correlations indicate a dominance of vegetation impact on soil moistures (growth depletes soil moisture). We used the *χ*^2^ test (and associated *p* values), the root mean square error of approximation (RMSEA), and an adjusted goodness-of-fit index (AGFI) to evaluate the fit of the established SEM models. The SEM analysis was implemented using the AMOS (version 21.0) software (Amos Development Corporation, Chicago, USA).

## Supplementary information

Supplementary Information

## Data Availability

All observation and model data that support the findings of this study are available as follows. The AVHRR GIMMS NDVI3g data are available at http://ecocast.arc.nasa.gov/data/pub/gimms/3g.v0/; The AVHRR GIMMS LAI3g data are available at http://cliveg.bu.edu/modismisr/lai3g-fpar3g.html; The FLUXCOM RS GPP product is available at http://www.fluxcom.org/; The ESA-CCI land-cover maps are available at http://maps.elie.ucl.ac.be/CCI/viewer/download.php; The climatic variables from the CRU v4.0.1 data are available at https://crudata.uea.ac.uk/cru/data/hrg/; The FLUXNET2015 EC measurements are available at https://fluxnet.fluxdata.org/2015/12/31/fluxnet2015-dataset-release/; The AmeriFlux EC measurements are available at https://ameriflux.lbl.gov/data/download-data/; The tree-ring width chronologies from the ITRDB V713 data are available at https://www.ncdc.noaa.gov/data-access/paleoclimatology-data/datasets/tree-ring/; the root-zone soil moisture from the GLEAM v3.2a data are available at https://www.gleam.eu/; model outputs generated by TRENDY v6 ecosystem models are available from Stephen Stich (s.a.sitch@exeter.ac.uk) or Pierre Friedlingstein (p.friedlingstein@exeter.ac.uk) upon request.

## Code availability

The processing MATLAB codes are available from the corresponding author upon request.
