# Supplementary material for: Seasonal biological carryover dominates northern vegetation growth
Source: Nat Commun. 2021 Feb 12;12:983. doi: 10.1038/s41467-021-21223-2 (PMC7881040; doi:10.1038/s41467-021-21223-2)
Supplement: Supplementary file 1 — Supplementary Information [file 41467_2021_21223_MOESM1_ESM.pdf]

Supplementary Information for

Seasonal biological carryover dominates northern vegetation growth

by Lian *et al.*

This PDF file contains:

Supplementary Tables 1-2

Supplementary Figures 1-18

Supplementary tables

Supplementary Table 1. Information of tree species included in the tree-ring database. The wood properties (RP=ring-porous wood, DP=diffuse-porous wood, NP=non-porous wood) of different tree species are derived from the wood database (https://www.wood-database.com/) or a search of the published literature.

Code	Species	Genus	Group	Wood type
ABAL	Abies alba Mill	Abies	Gymnosperms	NP
ABAM	Abies amabilis Dougl. ex Forbes	Abies	Gymnosperms	NP
ABBA	Abies balsamea (L.) Mill.	Abies	Gymnosperms	NP
ABBO	Abies borisii-regis Mattf.	Abies	Gymnosperms	NP
ABCE	Abies cephalonica Loud.	Abies	Gymnosperms	NP
ABCI	Abies cilicica (Ant. & Koischy) Carr.	Abies	Gymnosperms	NP
ABCO	Abies concolor (Gord. & Glend.) Lindl. ex Hildebr.	Abies	Gymnosperms	NP
ABDE	Abies pindrow	Abies	Gymnosperms	NP
ABFO	Abies forestii Rogers	Abies	Gymnosperms	NP
ABLA	Abies lasiocarpa	Abies	Gymnosperms	NP
ABMA	Abies magnifica A. Murr.	Abies	Gymnosperms	NP
ABNO	Abies nordmanniana (Slav.) Spach	Abies	Gymnosperms	NP
ABPI	Abies pindrow (Royle) Spach Himalayan	Abies	Gymnosperms	NP
ABPN	Abies pinsapo Boiss.	Abies	Gymnosperms	NP
ABRC	Abies recurvata Mast.	Abies	Gymnosperms	NP
ABSB	Abies spectabilIs	Abies	Gymnosperms	NP
ABSP	Abies Mill.	Abies	Gymnosperms	NP
ACRU	Acer rubrum L.	Acer	Angiosperms	DP
ACSH	Acer saccharum Marsh.	Acer	Angiosperms	DP
ADHO	Adesmia hornda Gill.	Adesmia	Angiosperms	RP

ADUS	Adesmia uspallatensis Gill.	Adesmia	Angiosperms	RP
AGAU	Agathis australis (D. Don) Lindley	Agathis	Gymnosperms	RP
ARAR	Araucaria araucana (Molina) K. Koch	Araucaria	Gymnosperms	NP
ATCU	Athrotaxis cupressoides D. Doo	Athrotaxis	Gymnosperms	NP
ATSE	Athrotaxis selaginoides D. Don	Athrotaxis	Gymnosperms	NP
AUCH	Austrocedrus chilensis (D. Don) Florin & Boutelje	Austrocedrus	Gymnosperms	NP
BEAK	Betula neoalaskana	Betula	Angiosperms	DP
BEER	Betula ermanii Cham.	Betula	Angiosperms	DP
BELE	Betula lenta L.	Betula	Angiosperms	DP
BEPU	Betula pubescens Ehm.	Betula	Angiosperms	DP
BEUT	Betula utilis D.Don	Betula	Angiosperms	DP
CABU	Canthium burttii	Canthium	Angiosperms	DP
CADE	Calocedrus decurrens	Libocedrus	Gymnosperms	NP
CARO	Callitris preissii Miq.	Callitris	Gymnosperms	NP
CASA	Castanea sativa Mill.	Castanea	Angiosperms	RP
CDAT	Cedrus atlantica (EndL) Manetti	Cedrus	Gymnosperms	NP
CDBR	Cedrus brevifolia Henry	Cedrus	Gymnosperms	NP
CDDE	Cedrus deodars (D. Don) G. Don	Cedrus	Gymnosperms	NP
CDLI	Cedrus libani A. Richard	Cedrus	Gymnosperms	NP
CEAN	Cedrela angustifolia Sesse & Mocino ex DC.	Cedrela	Angiosperms	RP
CESP	Cedrela Sp.	Cedrela	Angiosperms	RP
CHLA	Chamaecyparis lawsoniana	Chamaecypar is	Gymnosperms	NP
CHNO	Chamaecyparis nootkatensis (D. Don) Spach	Chamaecypar is	Gymnosperms	NP

CHOB	Chamaecyparis obtusa (Sieb. & Zucc.) Endl.	Chamaecyparis	Gymnosperms	NP
CMJA	Cryptomeria japonica (L. f.) D. Don	Cryptomeria	Gymnosperms	NP
CPBE	Carpinus betulus L.	Carpinus	Angiosperms	DP
CUCH	Cupressus chengiana	Cupressus	Gymnosperms	NP
CYGL	Carya glabra (Mill.) Sweet	Carya	Angiosperms	RP
CYOV	Carya ovata (MilL) K. Koch	Carya	Angiosperms	RP
DABI	Dacrydium biforme (Hook.) Pilger=Halocarpus	Dacrydium	Gymnosperms	NP
FAGR	Fagus grandifolia Ehrh.	Fagus	Angiosperms	DP
FASY	Fagus sylvatica	Fagus	Angiosperms	DP
FICU	Fitzroya cupressoides (Molina) Johnston	Fitzroya	Gymnosperms	NP
FOHO	Fokienia hodginsii	Fokienia	Gymnosperms	NP
FREX	Fraxinus excelsior L.	Fraxinus	Angiosperms	RP
HABI	Halocarpus biformis (Hook.) Quinn	Halocarpus	Gymnosperms	NP
HEHE	Hedera helix	Hedera	Angiosperms	DP
JGAU	Juglans australis Griseb.	Juglans	Angiosperms	DP
JUEX	Juniperus excelsa Bieb.	Juniperus	Gymnosperms	NP
JUFO	Juniperus foetidissima Willd.	Juniperus	Gymnosperms	NP
JUOC	Juniperus occidentalis Hook.	Juniperus	Gymnosperms	NP
JUOS	Juniperus osteosperma (Torr.) Little	Juniperus	Gymnosperms	NP
JUPH	Juniperus phoenicea L.	Juniperus	Gymnosperms	NP
JUPR	Juniperus przewalskii Kom.	Juniperus	Gymnosperms	NP
JURE	Juniperus recurva Such.-Harn. ex D. Don	Juniperus	Gymnosperms	NP
JUSC	Juniperus scopulorum Sarg.	Juniperus	Gymnosperms	NP
JUSP	Juniperus L.	Juniperus	Gymnosperms	NP
JUTI	Juniperus tibetica	Juniperus	Gymnosperms	NP
JUTU	Juniperus turkestanica Komar.	Juniperus	Gymnosperms	NP

JUVI	Juniperus virginians L.	Juniperus	Gymnosperms	NP
LADE	Larix decidua Mill	Larix	Gymnosperms	NP
LAGM	Larix gmelinii	Larix	Gymnosperms	NP
LAGR	Larix griffilhiana (Lindl. & Gord.) Carr.	Larix	Gymnosperms	NP
LALA	Larix laricina (Ou Roi) K. Koch	Larix	Gymnosperms	NP
LALY	Larix Iyalli	Larix	Gymnosperms	NP
LAOC	Larix occidentalis Nutt.	Larix	Gymnosperms	NP
LASI	Larix sibirica	Larix	Gymnosperms	NP
LASP	Larix Mill.	Larix	Gymnosperms	NP
LGCO	Lagarostrobus cclensoi (Hook.) C.J. Quinn=Dacrydium colensoi Hook.	Dacrydium	Gymnosperms	NP
LGFR	Lagsrostrobus frankliniiC.J. Quinn	Lagarostrobos	Gymnosperms	NP
LIBI	Libocedrus bidwillii Hook	Libocedrus	Gymnosperms	NP
LITU	Liriodendron tulipifera l.	Liriodendron	Angiosperms	DP
NOBE	Nothofagus betuloldes (Mirb.) Blume	Nothofagus	Angiosperms	DP
NOGU	Nothofsgus gunnii (Hook. F.) Oerst.	Nothofagus	Angiosperms	DP
NOME	Nothofagus menziesii (Hook. I.) Oerst.	Nothofagus	Angiosperms	DP
NOPU	Nothofagus pumilio	Nothofagus	Angiosperms	DP
NOSO	Nothofagus solandri	Nothofagus	Angiosperms	DP
PCAB	Picea abies(L.) Karst	Picea	Gymnosperms	NP
PCCH	Picea chihuahuana Martinez	Picea	Gymnosperms	NP
PCPU	Picea pungens Engelm	Picea	Gymnosperms	NP
PCEN	Picea engelmanni	Picea	Gymnosperms	NP
PCGL	Picea glauca	Picea	Gymnosperms	NP
PCGN	Picea glehnii (Fr. Schmidt) Mast.	Picea	Gymnosperms	NP
PCLI	Picea likiangensis (Franchet) Pritzel	Picea	Gymnosperms	NP
PCMA	Picea mariana	Picea	Gymnosperms	NP

PCOB	Picea obovata Ledeb.	Picea	Gymnosperms	NP
PCOM	Picea omorika (Panc.) Purk.	Picea	Gymnosperms	NP
PCOR	Picea orientalis (L.) Link	Picea	Gymnosperms	NP
PCRU	Picea rubens Sarg.	Picea	Gymnosperms	NP
PCSH	Picea shrenkiana Fisch. & Meyer	Picea	Gymnosperms	NP
PCSI	Picea sitchensis	Picea	Gymnosperms	NP
PCSM	Picea smithiana (Wall.) Boiss.	Picea	Gymnosperms	NP
PCSP	Picea A. Dietr.	Picea	Gymnosperms	NP
PCTI	Picea tienschanica Rupr.	Picea	Gymnosperms	NP
PHAS	Phyllocladus aspleniifolius (Labill.) Hook. f.	Phyllocladus	Gymnosperms	NP
PHGL	Phyllocladus glaucus carr.	Phyllocladus	Gymnosperms	NP
PHTR	Phyllocladus trichomanoides D. Don in Lamb.	Phyllocladus	Gymnosperms	NP
PIAL	Pinus albicaulis Engelm	Pinus	Gymnosperms	NP
PIAM	Pinus armandii Franchet	Pinus	Gymnosperms	NP
PIAR	Pinus arisiaia Engelm. in Parry & Engelm.	Pinus	Gymnosperms	NP
PIAZ	Pinus arizonica	Pinus	Gymnosperms	NP
PIBA	Pinus balfouriana Grev. & Ball. in A. Murr.	Pinus	Gymnosperms	NP
PIBN	Pinus bankasiana Lamb.	Pinus	Gymnosperms	NP
PIBR	Pinus brutia Ten	Pinus	Gymnosperms	NP
PICE	Pinus cembra L.	Pinus	Gymnosperms	NP
PICM	Pinus cembroides Zucc.	Pinus	Gymnosperms	NP
PICO	Pinus contorta Dougl. ex Loud.	Pinus	Gymnosperms	NP
PICU	Pinus culminicola	Pinus	Gymnosperms	NP
PIDE	Pinus densiflora Siab. & Zucco	Pinus	Gymnosperms	NP
PIEC	Pinus echinata Mill.	Pinus	Gymnosperms	NP

PIED	Pinus edulis	Pinus	Gymnosperms	NP
PIFL	Pinus flexilis James	Pinus	Gymnosperms	NP
PIGE	Pinus gerardiana Wall. exD. Don.	Pinus	Gymnosperms	NP
PIHA	Pinus halepensis Mill.	Pinus	Gymnosperms	NP
PIHE	Pinus heldrelchii Christ	Pinus	Gymnosperms	NP
PIHR	Pinus hartwegii	Pinus	Gymnosperms	NP
PIJE	Pinus jeffreyi Grev. & Balf. in A. Murr.	Pinus	Gymnosperms	NP
PIKE	Pinus kesiya Royle ex Gordon	Pinus	Gymnosperms	NP
PIKO	Pinus koraiensis Sieb. & Zucco	Pinus	Gymnosperms	NP
PILA	Pinus lambertiana Dougl.	Pinus	Gymnosperms	NP
PILE	Pinus leucodermis Ant.	Pinus	Gymnosperms	NP
PILO	Pinus longaeva D.K. Bailey	Pinus	Gymnosperms	NP
PIMA	Pinus massoniana	Pinus	Gymnosperms	NP
PIMG	Pinus mugo Turra	Pinus	Gymnosperms	NP
PIMK	Pinus merkusii Jungh. & De Vriese	Pinus	Gymnosperms	NP
PIMR	Pinus muricata D.Don	Pinus	Gymnosperms	NP
PIMU	Pinus mughus Scop=Pinus mugo Turra	Pinus	Gymnosperms	NP
PIMZ	Pinus montezumae	Pinus	Gymnosperms	NP
PINI	Pinus nigra Arnold	Pinus	Gymnosperms	NP
PIPA	Pinus palustris Mill.	Pinus	Gymnosperms	NP
PIPE	Pinus peuce Griseb.	Pinus	Gymnosperms	NP
PIPI	Pinus pinaster Ait.	Pinus	Gymnosperms	NP
PIPN	Pinus pinea L.	Pinus	Gymnosperms	NP
PIPO	Pinus ponderosa	Pinus	Gymnosperms	NP
PIPU	Pinus pungens Lamb.	Pinus	Gymnosperms	NP
PIRE	Pinus resinosa Ait.	Pinus	Gymnosperms	NP
PIRI	Pinus rigida Mill.	Pinus	Gymnosperms	NP
PIRO	Pinus roxburghii Sarg.	Pinus	Gymnosperms	NP

PISF	Pinus strobiformis Engelm. in Wisliz.	Pinus	Gymnosperms	NP
PISI	Pinus sibirica Du Tour	Pinus	Gymnosperms	NP
PISP	Pinus L.	Pinus	Gymnosperms	NP
PIST	Pinus strobus L	Pinus	Gymnosperms	NP
PISY	Pinus sylvestris	Pinus	Gymnosperms	NP
PITA	Pinus taeda L.	Pinus	Gymnosperms	NP
PITB	Pinus tabulaeformis Carr.	Pinus	Gymnosperms	NP
PIUN	Pinus uncinata Mill. ex Mirb. in Buffon	Pinus	Gymnosperms	NP
PIVI	Pinus virginiana Mill.	Pinus	Gymnosperms	NP
PIWA	Pinus wallichiana A.B. Jackson	Pinus	Gymnosperms	NP
PLRA	Platanus racemosa	Platanus	Angiosperms	DP
PPDE	Populus deltoides Bartr. ex Marsh.	Populus	Angiosperms	DP
PPGR	Populus grandidentata Michx.	Populus	Angiosperms	DP
PPSP	Populus L.	Populus	Angiosperms	DP
PPTM	Populus tremuloides	Populus	Angiosperms	DP
PPTR	Populus tremuloides Michx.	Populus	Angiosperms	DP
PROS	Prosopis L.	Prosopis	Angiosperms	DP
PSMA	Pseudotsuga macrocarpa (Vasey) Mayr	Pinus	Gymnosperms	NP
PSME	Pseudotsuga menziesii	Pseudotsuga	Gymnosperms	NP
PTAN	Pterocarpus angolensis DC.	Pterocarpus	Angiosperms	DP
PTLE	Pistacia lentiscus	Pistacia	Angiosperms	RP
QUAL	Quercus alba L.	Quercus	Angiosperms	RP
QUCA	Quercus canariensis Willd.	Quercus	Angiosperms	RP
QUCE	Quercus cerris L.	Quercus	Angiosperms	RP
QUCO	Quercus coccinea Muenchh.	Quercus	Angiosperms	RP
QUDG	Quercus douglasii Hook and Am.	Quercus	Angiosperms	RP
QUFA	Quercus falcata Michx.	Quercus	Angiosperms	RP
QUFG	Quercus faginea Lam.	Quercus	Angiosperms	RP

QUFR	Quercus frainetto Ten.	Quercus	Angiosperms	RP
QUHA	Quercus hartwissiana	Quercus	Angiosperms	RP
QUKE	Quercus kelloggii Newb.	Quercus	Angiosperms	RP
QULO	Quercus lobata Nee	Quercus	Angiosperms	RP
QULY	Quercus Iyrata Walt.	Quercus	Angiosperms	RP
QUMA	Quercus macrocarpa	Quercus	Angiosperms	RP
QUMC	Quercus macrolepis	Quercus	Angiosperms	RP
QUMO	Quercus mongolica Fisch. Ex Turcz.	Quercus	Angiosperms	RP
QUMU	Quercus muehlenoorgii Engelm.	Quercus	Angiosperms	RP
QUPA	Quercus palustris Muenchh.	Quercus	Angiosperms	RP
QUPE	Quercus petraea	Quercus	Angiosperms	RP
QUPR	Quercus prinus L.	Quercus	Angiosperms	RP
QUPU	Quercus pubescens Willd.	Quercus	Angiosperms	RP
QURO	Quercus robur	Quercus	Angiosperms	RP
QURU	Quercus rubra L.	Quercus	Angiosperms	RP
QUSH	Quercus shumardii Buck1.	Quercus	Angiosperms	RP
QUSP	Quercus L.	Quercus	Angiosperms	RP
QUST	Quercus stellata	Quercus	Angiosperms	RP
QUVE	Quercus velutina Larn.	Quercus	Angiosperms	RP
SALA	Salix lanata L.	Salix	Angiosperms	DP
SAPC	Salix phylicifolia	Salix	Angiosperms	DP
TABA	Taxus baccata L.	Taxus	Gymnosperms	NP
TADI	Taxodium distichum (L.) Rich.	Taxodium	Gymnosperms	NP
TAMU	Taxodium mucronatum	Taxodium	Gymnosperms	NP
TEGR	Tectona grandis L. f.	Tectona	Angiosperms	RP
THOC	Thuja accidentalis L.	Thuja	Gymnosperms	NP
THPL	Thuja plicata Donn ex D. Don	Thuja	Gymnosperms	NP
TICO	Tilia cordata Mill.	Tilia	Angiosperms	DP

TSCA	Tsuga canadensis	Tsuga	Gymnosperms	NP
TSCR	Tsuga caroliniana Engelm.	Tsuga	Gymnosperms	NP
TSDU	Tsuga dumosa (D.Don) Eichl.	Tsuga	Gymnosperms	NP
TSHE	Tsuga heterophylla (Raf.) Sarg.	Tsuga	Gymnosperms	NP
TSME	Tsuga mertensiana	Tsuga	Gymnosperms	NP
ULSP	Ulmus L.	Ulmus	Angiosperms	RP
VIKE	Vitex Kiniensis	Vitex	Angiosperms	DP
WICE	Widdringtonia cedarbergensis J.A. Marsh	Clanwilliam cedar	Gymnosperms	NP

Supplementary Table 2. Details of the 16 TRENDY terrestrial ecosystem models used in this study.

Model Name	Model name abbreviation	Spatial resolution	Reference
Community Atmosphere Biosphere Land Exchange	CABLE	0.5° × 0.5°	(1)
Community Land Model version 4.5	CLM4.5	1.25° × 0.9375°	(2)
Organizing Carbon and Hydrology in Dynamic Ecosystems	ORCHIDEE	0.5° × 0.5°	(3)
ORCHIDEE aMeliorated Interactions between Carbon and Temperature	ORCHIDEE-MICT	1° × 1°	(4)
Vegetation Integrative Simulator for Trace gases	VISIT	0.5° × 0.5°	(5)
Jena Scheme for Biosphere-Atmosphere Coupling in Hamburg	JSBACH	1.875° × 1.875°	(6)
Lund-Potsdam-Jena General Ecosystem Simulator	LPJ-GUESS	0.5° × 0.5°	(7)
Land Surface Processes and Exchanges model of the University of Bern	LPX-Bern	1° × 1°	(8)
Lund-Potsdam-Jena Wald Schnee und Landscaft version	LPJ-wsl	0.5° × 0.5°	(9)
The Joint UK Land Environment Simulator	JULES	1.875° × 1.25°	(10, 11)

Integrated Science Assessment Model	ISAM	0.5° × 0.5°	(12)
Sheffield Dynamic Global Vegetation Model	SDGVM	1° × 1°	(13)
Vegetation Global Atmosphere Soil	VEGAS	0.5° × 0.5°	(14)
ORCHIDEE-CN	OCN	3.75° × 2.5°	(15)
Canadian Land Surface Scheme-Canadian Terrestrial Ecosystem Model	CLASS-CTEM	2.8125° × 2.8125°	(16)
Dynamic Land Ecosystem Model	DLEM	0.5° × 0.5°	(17)

References

1. Wang, Y. P., Law, R. M. & Pak, B. A global model of carbon, nitrogen and phosphorus cycles for the terrestrial biosphere. *Biogeosciences* **7**, 2261-2282 (2010).
2. Oleson, K. W. *et al.* Technical description of version 4.0 of the Community Land Model (CLM). *NCAR/TN-503+STR NCAR Tech. Note* 266 (2013). doi:10.5065/D6FB50WZ
3. Krinner, G. *et al.* A dynamic global vegetation model for studies of the coupled atmosphere-biosphere system. *Global Biogeochem. Cycles* **19**, 1-33 (2005).
4. Guimberteau, M. *et al.* ORCHIDEE-MICT (v8.4.1), a land surface model for the high latitudes: model description and validation. *Geosci. Model Dev.* **11**, 121-163 (2018).
5. Kato, E., Kinoshita, T., Ito, A., Kawamiya, M. & Yamagata, Y. Evaluation of spatially explicit emission scenario of land-use change and biomass burning using a process-based biogeochemical model. *J. Land Use Sci.* **8**, 104-122 (2013).
6. Kaminski, T. *et al.* The BETHY/JSBACH carbon cycle data assimilation system: experiences and challenges. *J. Geophys. Res. Biogeosciences* **118**, 1414-1426 (2013).
7. Smith, B., Prentice, I. C. & Sykes, M. T. Representation of vegetation dynamics in the modelling of terrestrial ecosystems: comparing two contrasting approaches within European climate space. *Glob. Ecol. Biogeogr.* **10**, 621-637 (2001).
8. Stocker, B. D. *et al.* Multiple greenhouse-gas feedbacks from the land biosphere under future climate change scenarios. *Nat. Clim. Change* **3**, 666-672 (2013).
9. Sitch, S. *et al.* Evaluation of ecosystem dynamics, plant geography and terrestrial carbon cycling in the LPJ dynamic global vegetation model. *Glob. Change Biol.* **9**, 161-185 (2003).
10. 10. Best, M. J. *et al.* The Joint UK Land Environment Simulator (JULES), model description – Part 1: Energy and water fluxes. *Geosci. Model Dev.* **4**, 677–699 (2011).
11. Clark, D. B. *et al.* The Joint UK Land Environment Simulator (JULES), model description – Part 2: Carbon fluxes and vegetation dynamics. *Geosci. Model Dev.* **4**, 701-722 (2011).

12. Jain, A. K. & Yang, X. Modeling the effects of two different land cover change data sets on the carbon stocks of plants and soils in concert with CO_2 and climate change. *Global Biogeochem. Cycles* **19**, GB2015 (2005).
13. Woodward, F. I. & Lomas, M. R. Vegetation dynamics – simulating responses to climatic change. *Biol. Rev.* **79**, 643-670 (2004).
14. Zeng, N., Mariotti, A. & Wetzel, P. Terrestrial mechanisms of interannual CO_2 variability. *Global Biogeochem. Cycles* **19**, 1-15 (2005).
15. Zaehle, S. *et al.* Carbon and nitrogen cycle dynamics in the O-CN land surface model: 2. Role of the nitrogen cycle in the historical terrestrial carbon balance. *Global Biogeochem. Cycles* **24**, GB1006 (2010).
16. Huang, S. *et al.* Analysis of nitrogen controls on carbon and water exchanges in a conifer forest using the CLASS-CTEMN+ model. *Ecol. Modell.* **222**, 3743-3760 (2011).
17. Tian, H. *et al.* Century-scale responses of ecosystem carbon storage and flux to multiple environmental changes in the Southern United States. *Ecosystems* **15**, 674-694 (2012).

Supplementary figures

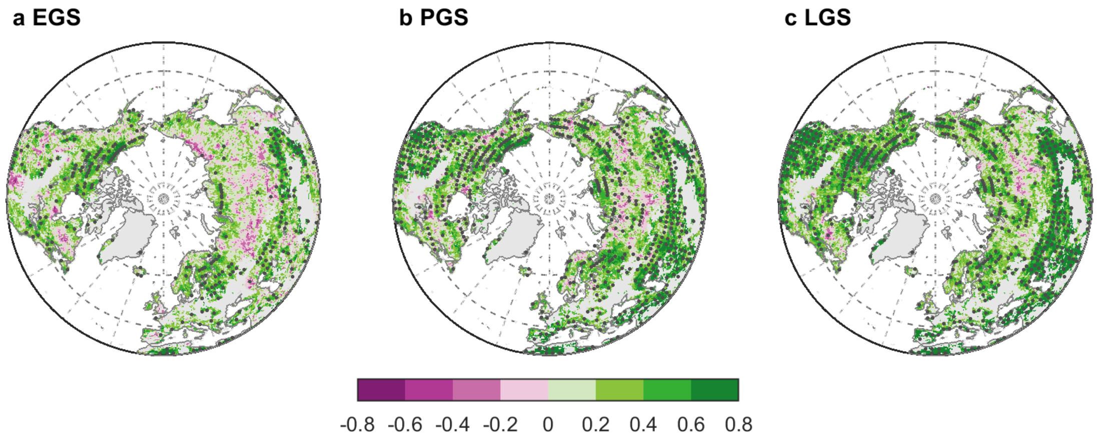

Supplementary Figure 1. Spatial distribution of the partial correlations between NDVI of each season and that of the preceding season. Correlation patterns are shown for EGS (**a**), PGS (**b**) and LGS (**c**). The partial correlations remove the covarying effects of the climatic variables of the focused season and its preceding season. For preceding-season NDVI of EGS we use the NDVI of the preceding LGS instead of the preceding winter. Black dots indicate statistically significant correlations at the 95% confidence level.

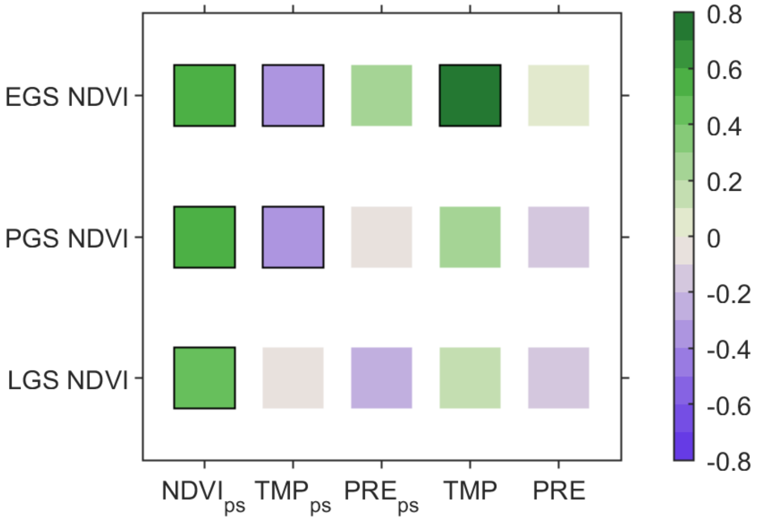

Supplementary Figure 2. Partial correlations between detrended anomalies of NDVI and that of its different factors. This is same as Fig. 1a, except that all variables were detrended before performing partial correlation analysis. As for Fig. 1a, partial correlation coefficients are between 35-year seasonal NDVI time series and concurrent climatic factors (TMP and PRE), and climatic factors (TMPps and PREps) and NDVI (NDVIps) of the preceding season. The subscript ps denotes values for the immediately preceding season, except that ps for EGS NDVI refers to NDVI of the preceding LGS. Squares with black outline show statistically significant correlations at the 95% confidence level.

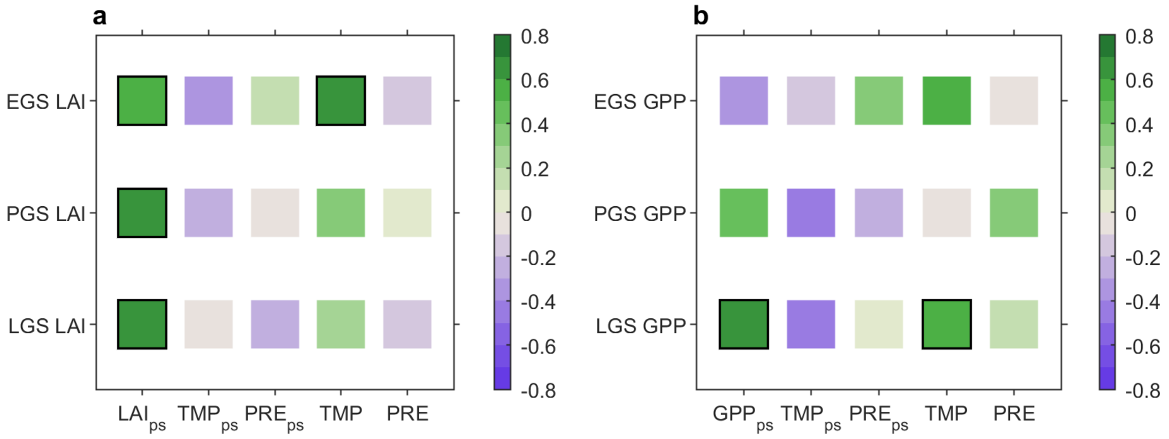

Supplementary Figure 3. Partial correlations between anomalies of vegetation growth and that of its different factors. This is same as Fig. 1a, except that vegetation growth is here based on GIMMS LAI (**a**) and FLUXCOM-RS GPP (**b**). As for Fig 1a, partial correlation coefficients are between seasonal LAI (or GPP) time series and concurrent climatic factors (TMP and PRE), and climatic factors (TMPps and PREps) and LAI (or GPP) of the preceding season (LAIps or GPPps). The subscript ps denotes values for the immediately preceding season, except that ps for EGS LAI (or GPP) refers to LAI (or GPP) of the preceding LGS. Squares with black outline show statistically significant correlations at the 95% confidence level.

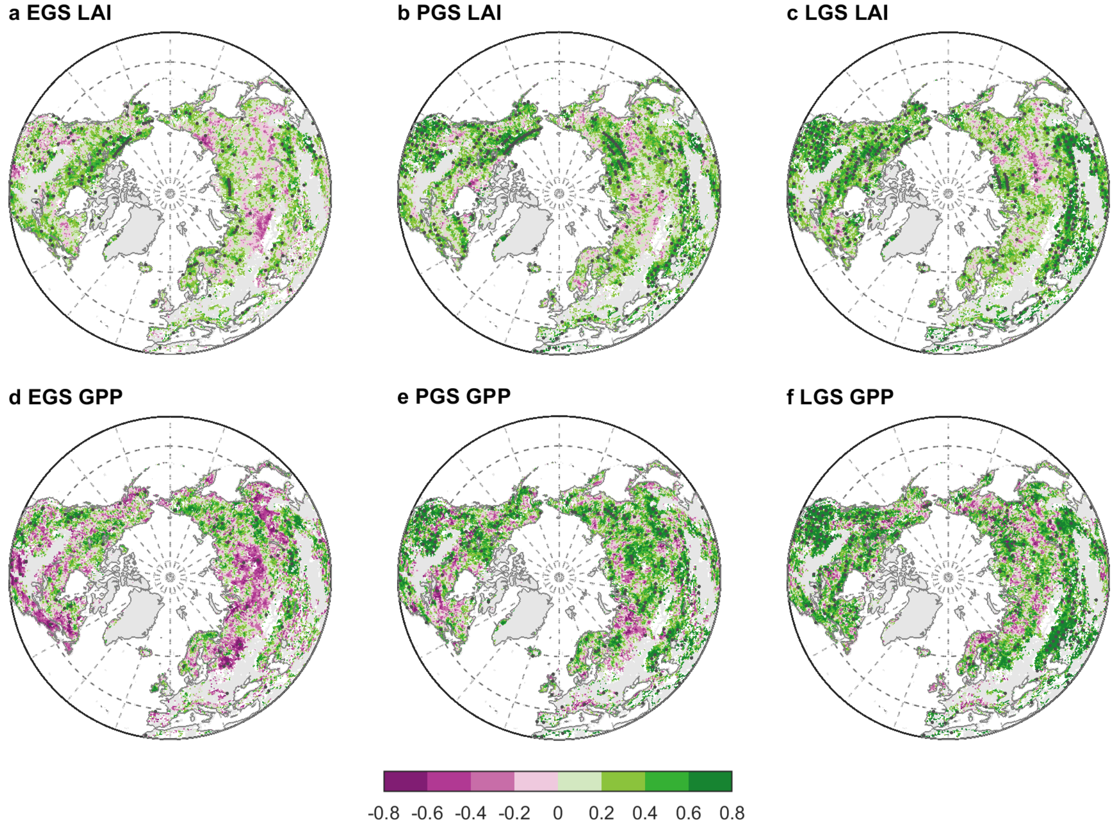

Supplementary Figure 4. Spatial distribution of the partial correlations between GIMMS LAI (**a-c**) or FLUXCOM-RS GPP (**d-f**) of each season and that of the preceding season. The partial correlations remove the covarying effects of the climatic variables of the focused season and its preceding season. For preceding-season LAI (or GPP) of EGS we use the LAI (or GPP) of the preceding LGS instead of the preceding DS. Black dots indicate statistically significant correlations at the 95% confidence level.

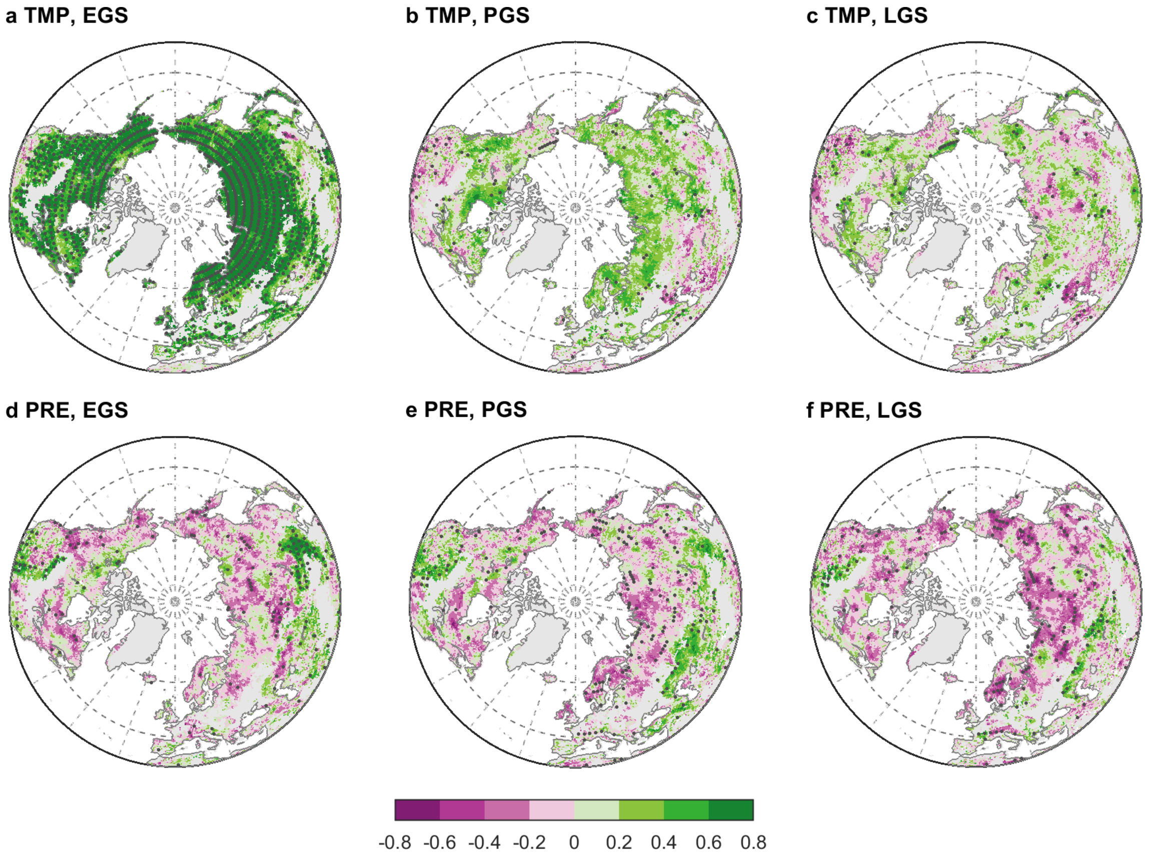

Supplementary Figure 5. Spatial distribution of the partial correlations between NDVI of each season and the concurrent climate. Correlation patterns are shown for EGS (**a**, **d**), PGS (**b**, **e**) and LGS (**c**, **f**), and for correlation with concurrent temperature (**a-c**) and precipitation (**d-f**). The partial correlations remove the covarying effects of both preceding-season NDVI and preceding-season climate. For preceding-season NDVI of EGS we use NDVI of the preceding LGS instead of the preceding winter. Black dots indicate statistically significant correlations at the 95% confidence level.

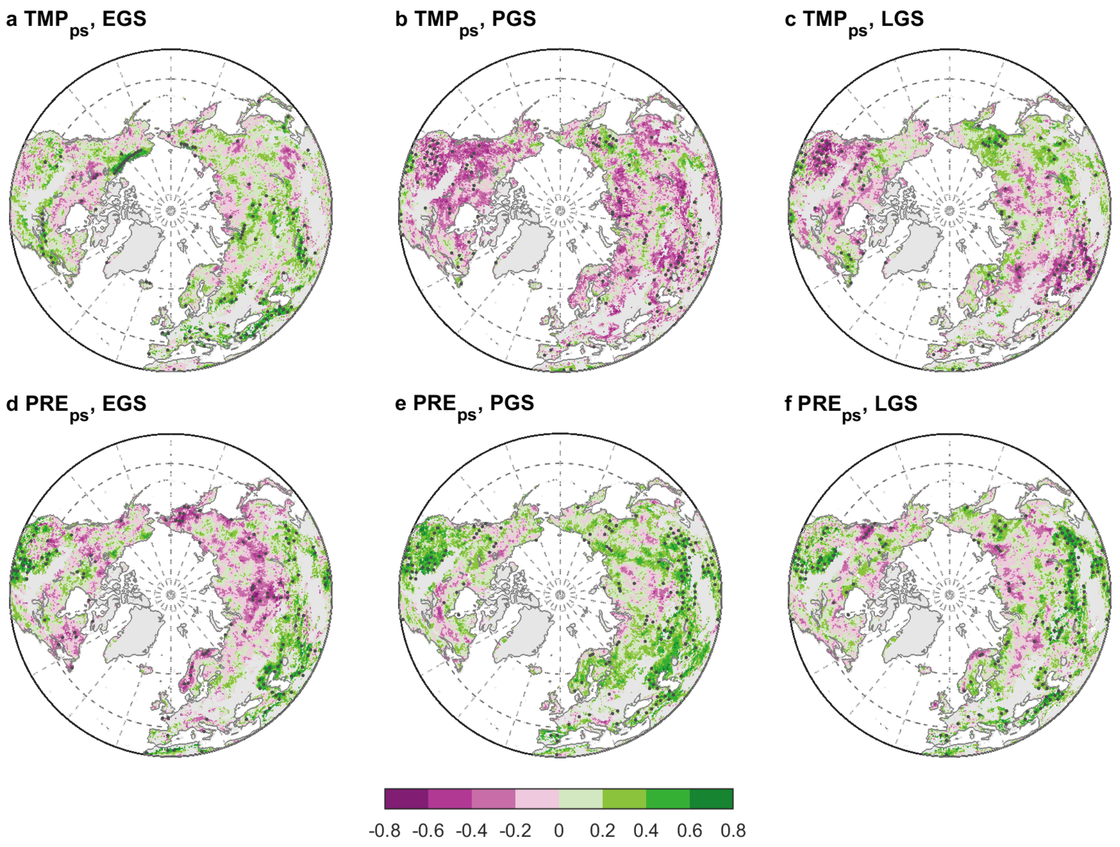

Supplementary Figure 6. Spatial distribution of the partial correlations between NDVI of each season and climate of the preceding season. Correlation patterns are shown for EGS (**a**, **d**), PGS (**b**, **e**) and LGS (**c**, **f**), and for correlation with preceding-season temperature (**a-c**) and precipitation (**d-f**). The partial correlations remove the covarying effects of climate for a season and NDVI for the preceding season. For preceding-season NDVI of EGS we use NDVI of the preceding LGS instead of the preceding winter. Black dots indicate statistically significant correlations at the 95% confidence level.

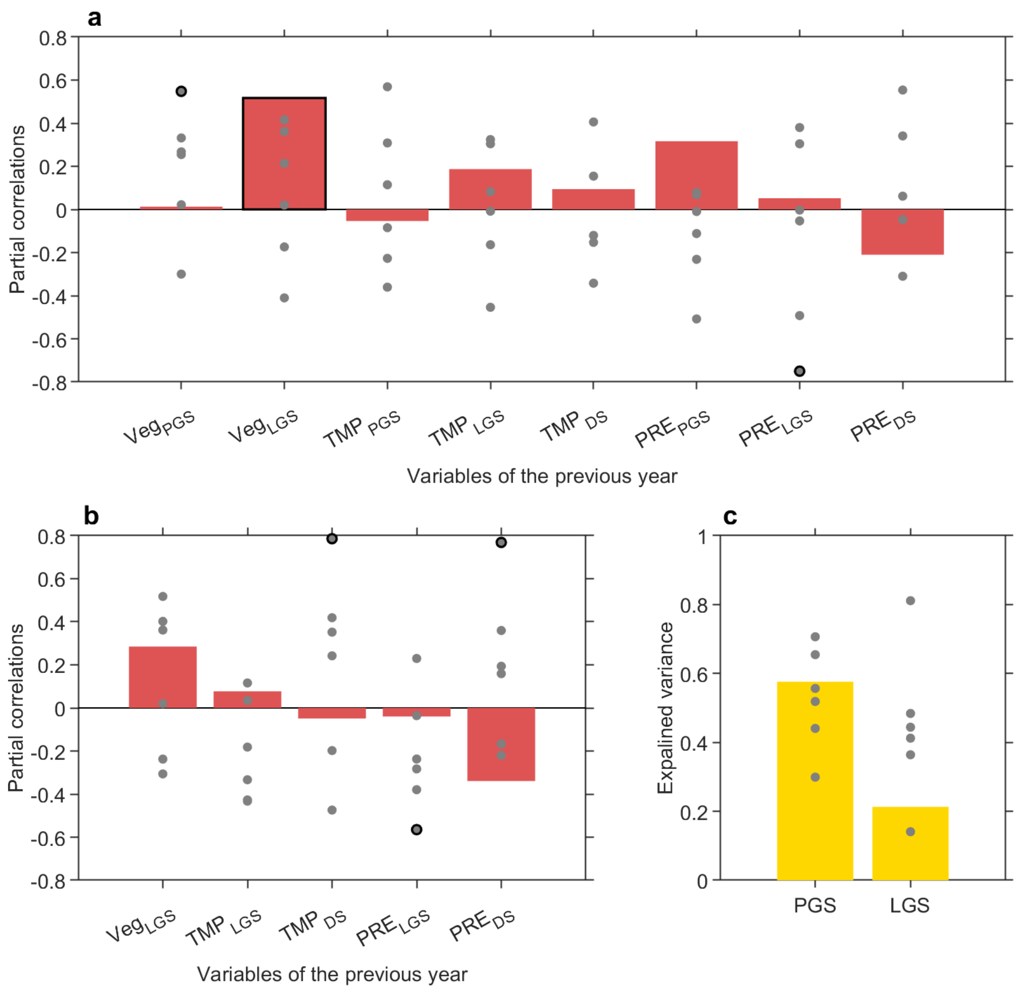

Supplementary Figure 7. Impact of vegetation growth and climatic factors of the previous year on current vegetation growth. Partial correlation coefficients of the residuals of seasonal vegetation growth (**a** for PGS and **b** for LGS) and driving factors of the previous year. The residuals represent the term ε in Eqn. 1, after accounting for the influence of factors of the current season, and that of the previous season for the current year. **c**, the fraction of explained variance of the residuals by all the driving factors. In all panels, bars show the results of satellite-derived NDVI data, and dots show that of GPP measurements from 6 Ameriflux sites (other sites with less than 5 degrees of freedom were removed for this analysis). In panels **a** and **b**, bars and dots edged with black indicate statistically significant correlations at the 95% confidence level.

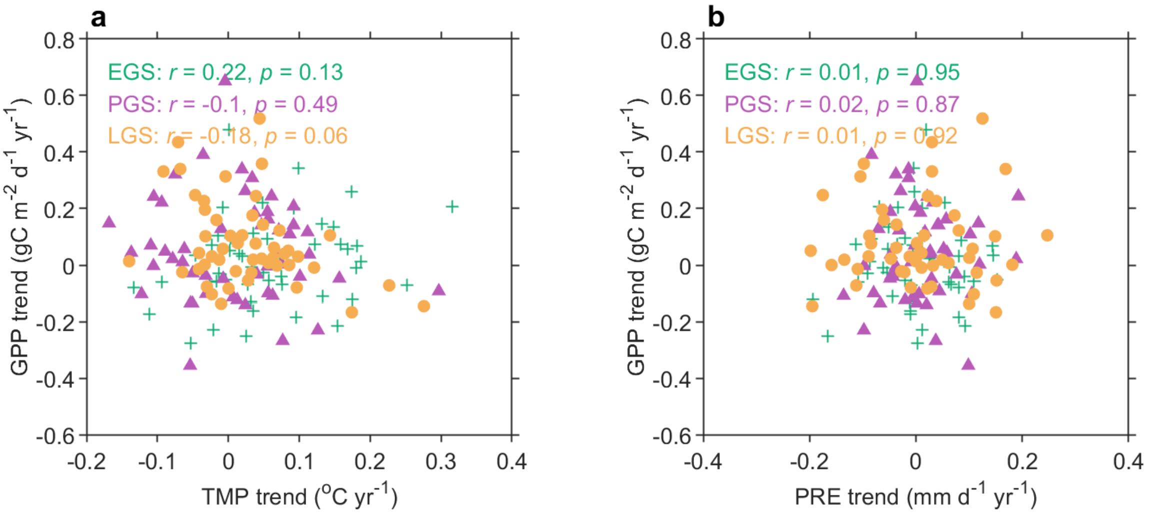

Supplementary Figure 8. Site-observed responses of GPP to concurrent climate. **a**, Scatterplot of the trend of GPP against that of air temperature across flux sites for EGS, PGS, and LGS. **b**, Scatterplot of the trend of GPP against that of precipitation across flux sites for EGS, PGS, and LGS. Linear regressions are shown in both panels and for each season (straight lines), along with related statistics as annotated.

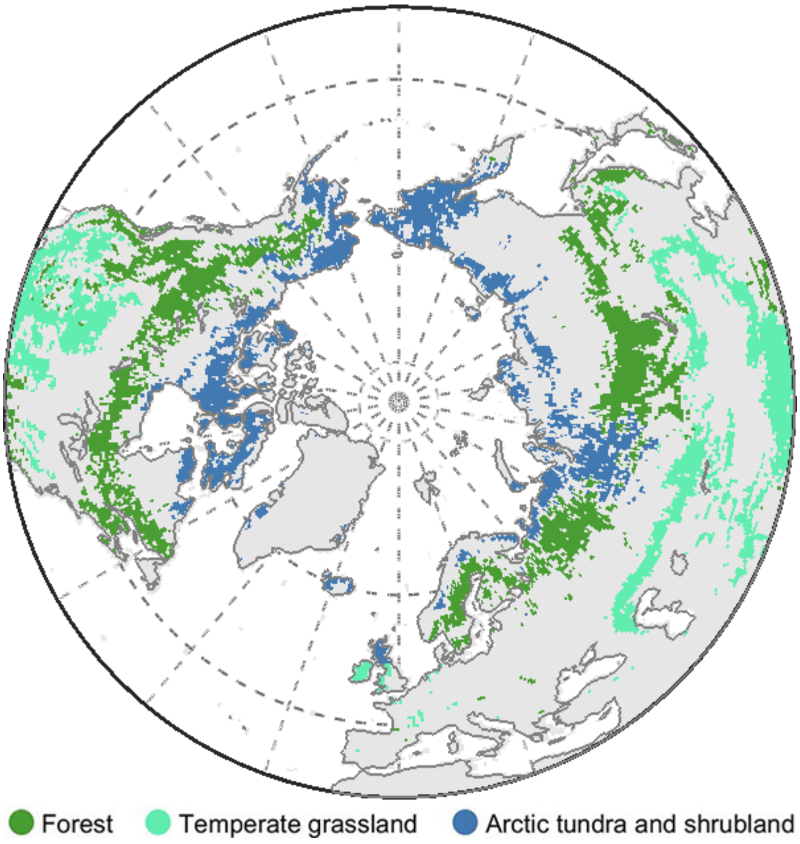

Supplementary Figure 9. Spatial distribution of the three major vegetation types (forest, temperate grassland, arctic tundra and shrubland) based on ESA-CCI land cover maps. Only grid cells where the dominant vegetation type occupies >60% of the grid area over the 1992-2016 period are shown and considered in the SEM analyses.

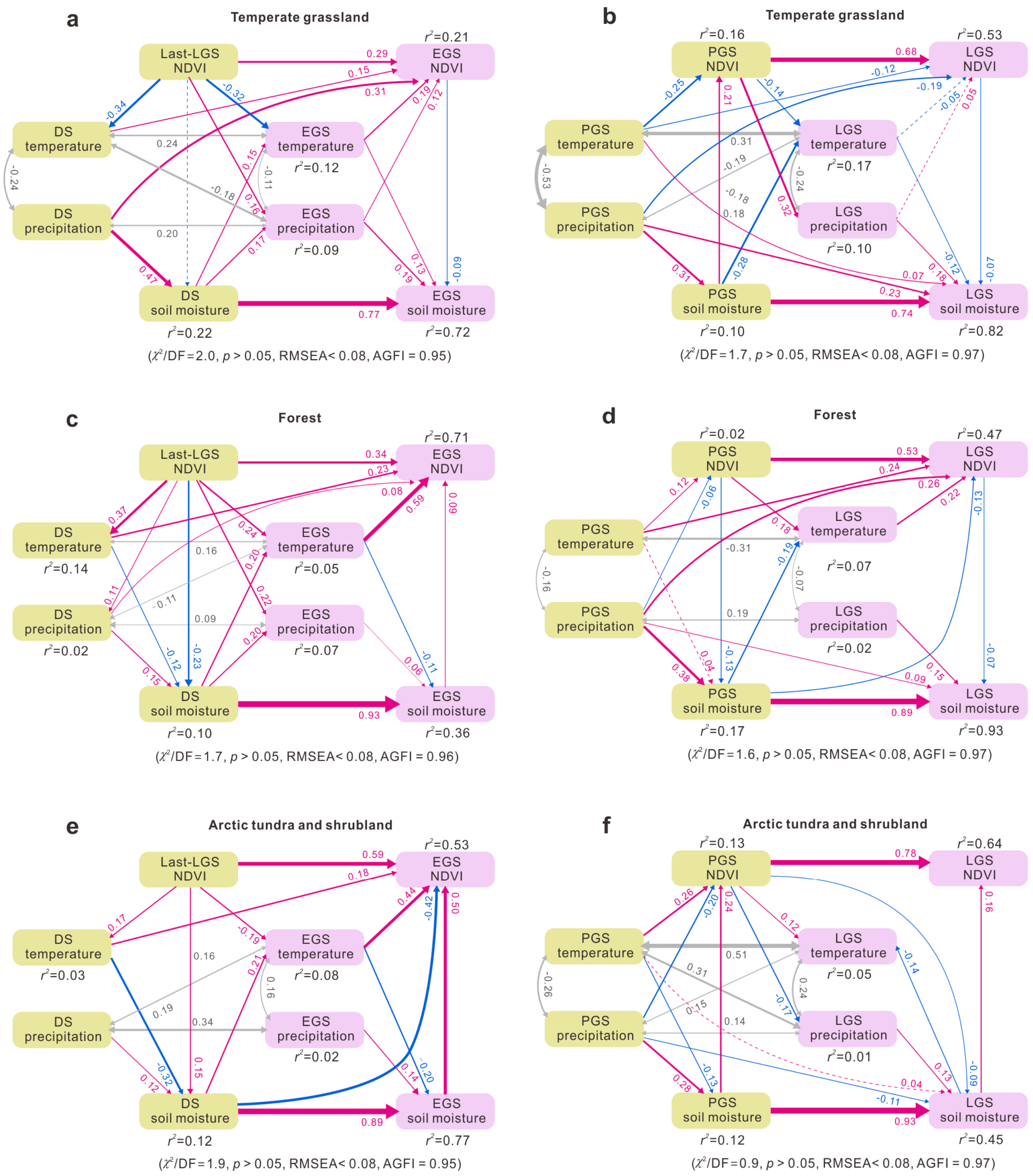

Supplementary Figure 10. Identical diagram as Fig 3, but here presenting the direct and indirect (climate or VGC) effects instead for seasons of EGS NDVI (a, c, e) and LGS NDVI (b, d, f).

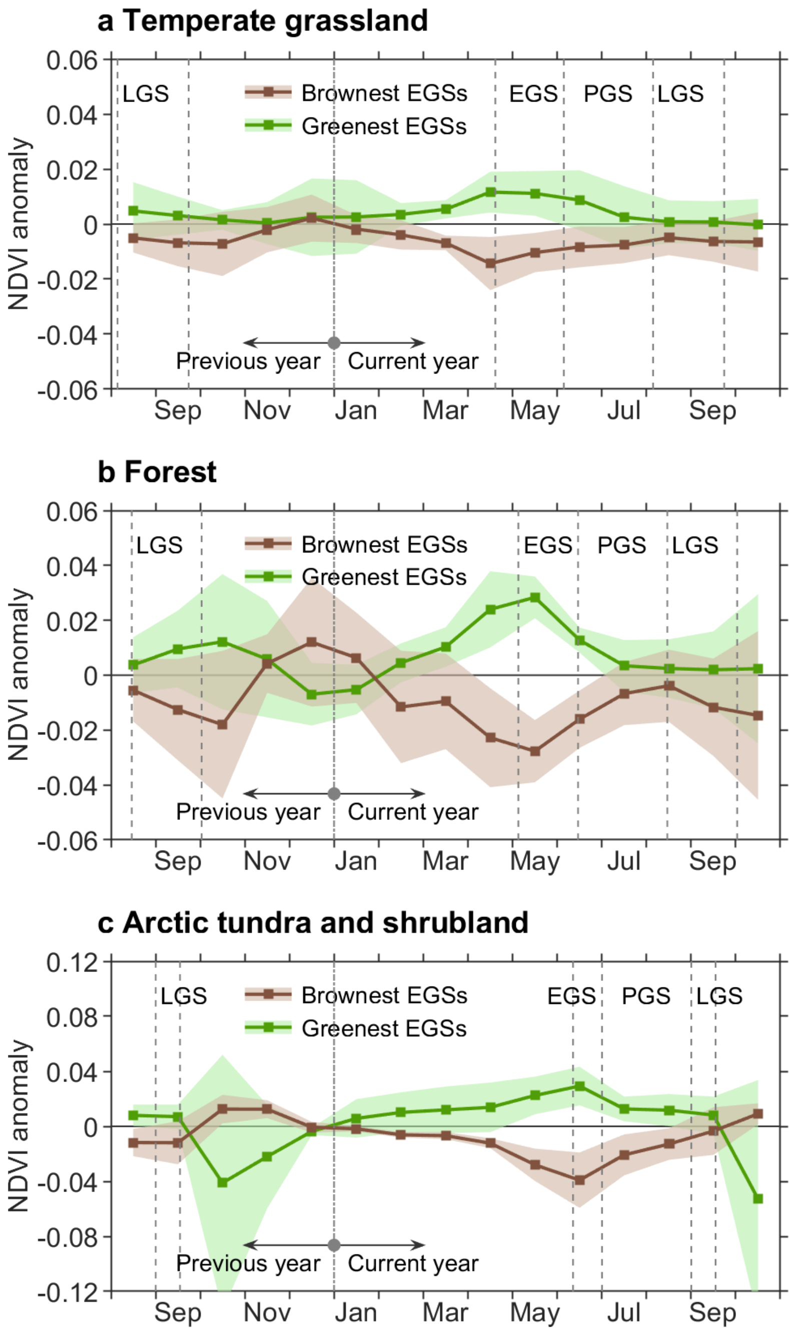

Supplementary Figure 11. Monthly NDVI anomalies for years with the greenest and brownest EGSs. Anomalies presented are calculated by compositing the eight years with the largest (greenest) and the smallest (brownest) NDVI values during 1982-2016 relative to the climatological mean of this period. This maximum and minimum composition analysis was conducted for Northern Latitude regions dominated by temperate grassland (Tibetan Plateau excluded) (**a**), forest (**b**) and arctic tundra and shrubland (**c**) (see Supplementary Fig. 9). The shadings show the 1-S.D. of the eight-year compositions. The vertical dashed lines give definitions of the seasonal timeframes used (see Methods).

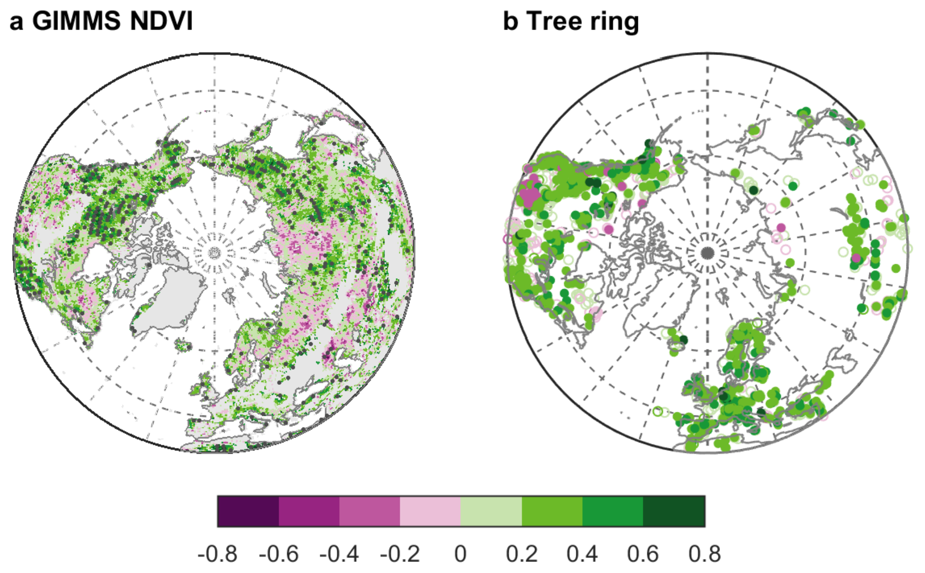

Supplementary Figure 12. Spatial distribution of the partial correlations between NDVI (**a**), or tree ring width (**b**), of each year and that of the preceding year. The partial correlations enable removal of any co-varying effects of climatic variables of a year and its preceding year. In both panels, filled dots represent statistically significant locations at the 95% confidence level. Note that all variables are detrended before performing partial correlation analysis.

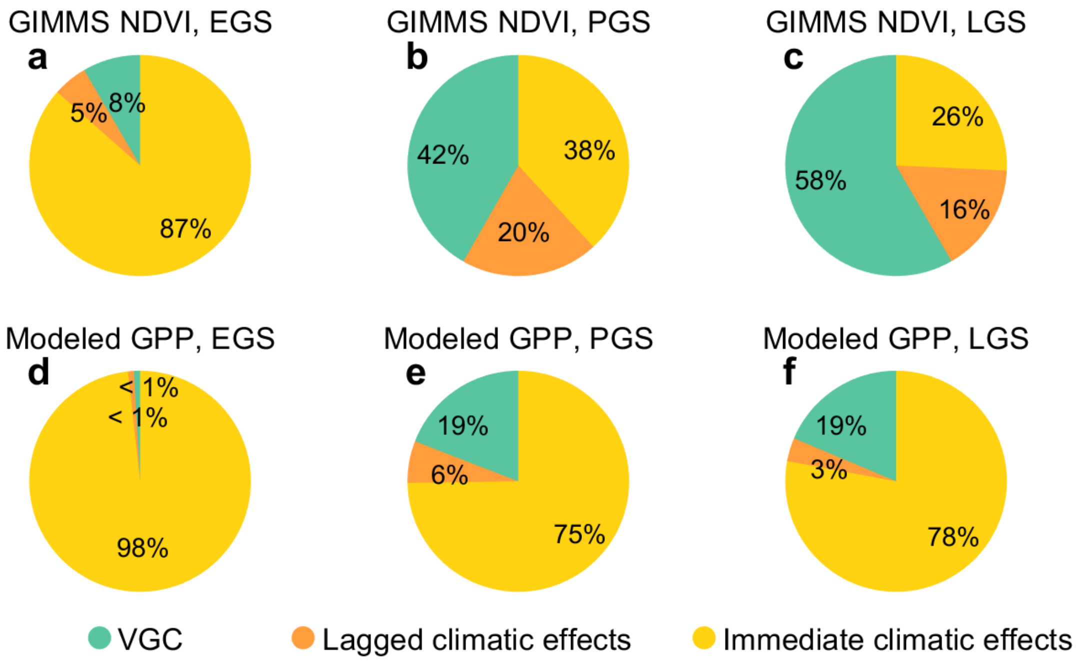

Supplementary Figure 13. Spatial coverage of the dominant drivers of vegetation growth and productivity for each season. The spatial coverage of each driver of vegetation growth (NDVI) and productivity (GPP) is represented as an overall percentage of the vegetated and non-agricultural areas of the Northern Hemisphere (30-90°N). The percentages in the pie plots are given as green for VGC, orange for preceding-season climate, and yellow for concurrent climate. This diagram summarises the statistics of patterns shown in Fig. 5.

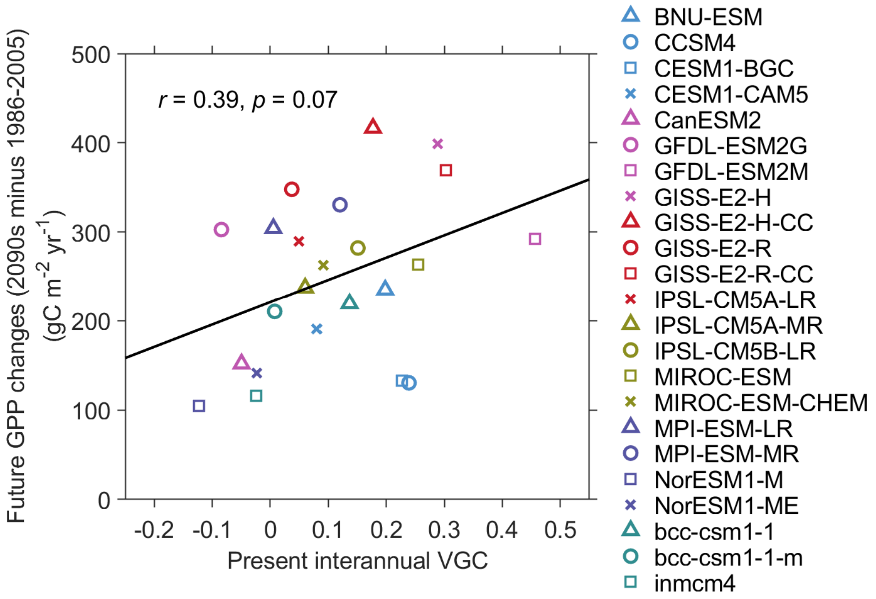

Supplementary Figure 14. Relationship across Earth System Models between present-day VGC values and future changes to GPP. Cross-model relationship between projected GPP increase for 2091-2100 relative to 1986-2005, and modeled present interannual VGC values, over the Northern Hemisphere. Model predictions are based on projections by Earth System Models in the Coupled Model Intercomparison Project Phase 5 (CMIP5) under the RCP4.5 scenario. The interannual VGC was calculated as the partial correlations between GPP of each year and the preceding year, while controlling for the co-varying effects of climatic variables of a year and its preceding year (all variables detrended), during the period 1976-2005.

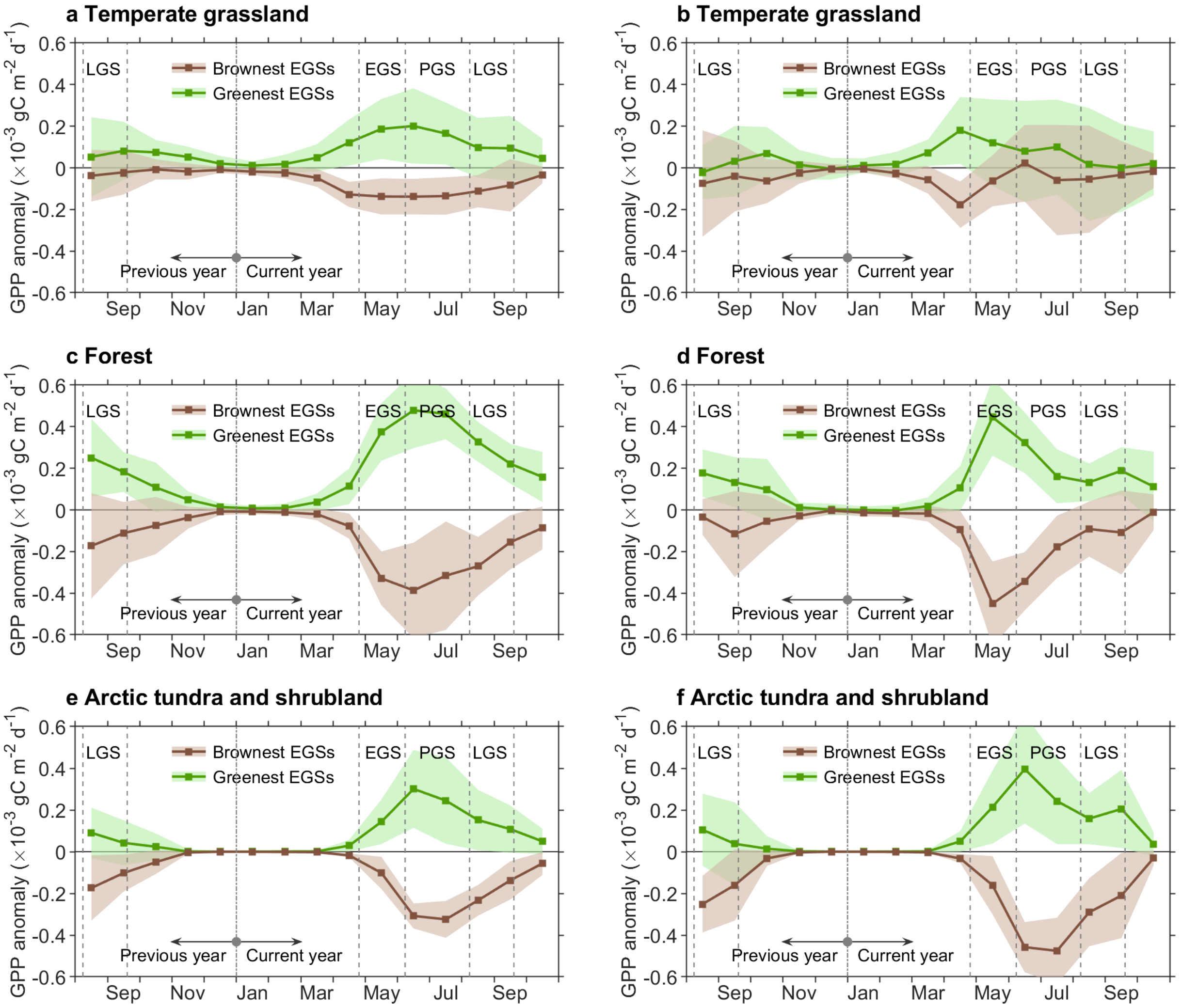

Supplementary Figure 15. Monthly GPP anomalies for models with contrasting performances of VGC. Diagram has same format as Supplementary Figure 11, but based on model simulations. Comparison of the seasonal trajectories of GPP for years with the greenest and brownest EGSs, and for the three models that best identify EGS-to-PGS VGC (ISAM, OCN and DLEM, based on Fig. 5f) (**a**, **c**, **e**) and that least capture EGS-to-PGS VGC (LPX-Bern, LPJ-wsl and CLASS-CTEM, based on Fig. 5f) (**b**, **d**, **f**). These NDVI anomalies were calculated by compositing the eight years with the largest (greenest) and the smallest (brownest) NDVI values during 1982-2016 relative to the climatological mean of this period. This maximum (minimum) composition analysis was conducted for regions dominated by temperate grassland (Tibetan Plateau excluded) (**a**, **b**), forest (**c**, **d**) and arctic tundra and shrubland (**e**, **f**) (see Supplementary Fig. 9). The shadings show the S.D. of the eight-year compositions. The vertical dashed lines give definitions of the seasonal timeframes used (see Methods).

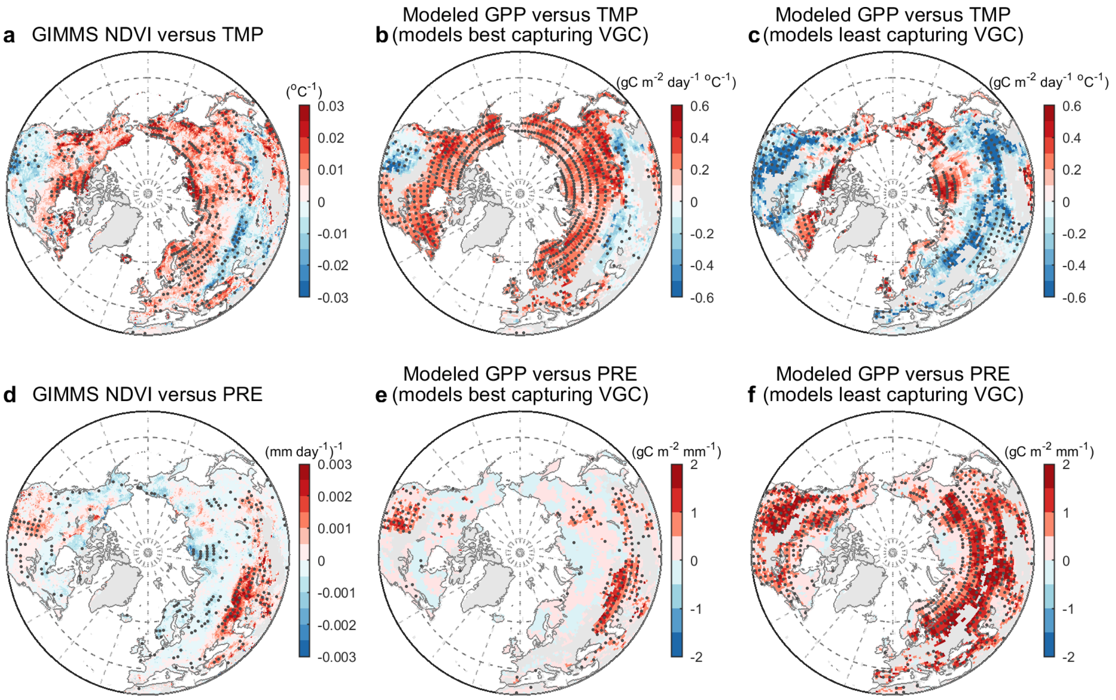

Supplementary Figure 16. Comparison of climatic sensitivities of observed PGS NDVI and modeled PGS GPP. Spatial distribution of the sensitivities of observed PGS productivity (indicated by NDVI) to PGS temperature (**a**) and PGS precipitation (**d**). Panels (**b**) and (**e**) are identical to those of (**a**) and (**b**) respectively, but using GPP estimates from three models that best identify EGS-to-PGS VGC (ISAM, OCN and DLEM, based on Fig. 5f). Modelled GPP is taken as representative of NDVI. Panels (**c**) and (**f**) are identical to those of (**b**) and (**e**) respectively, but for the three models that least capture EGS-to-PGS VGC (LPX-Bern, LPJ-wsl and CLASS-CTEM, based on Fig. 5f). In all panels, black dots indicate statistically significant regions at the 95% confidence level.

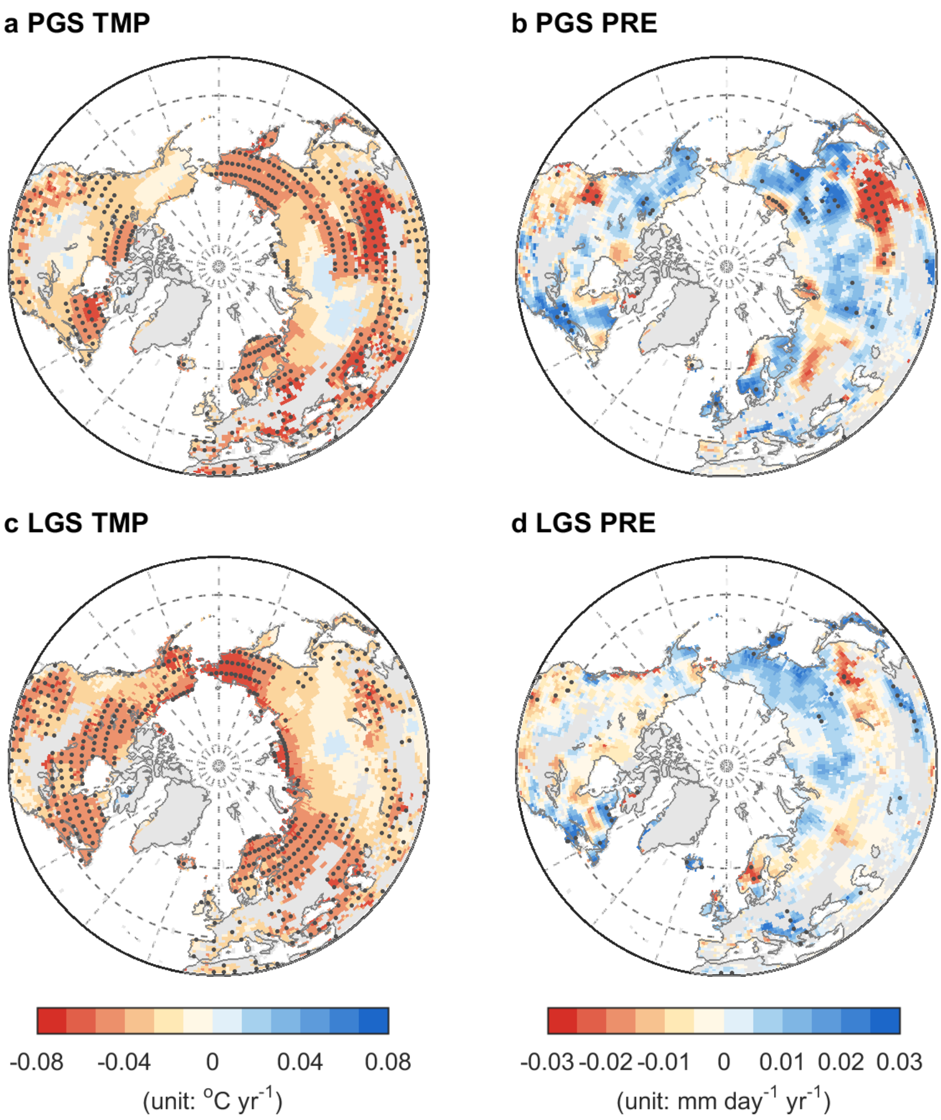

Supplementary Figure 17. Spatial distribution of the observed trend of temperature and precipitation. Linear trends of **(a)** PGS temperature, **(b)** PGS precipitation, **(c)** LGS temperature and **(d)** LGS precipitation, calculated for 1982-2016. Black dots indicate statistically significant trends at the 95% confidence level.

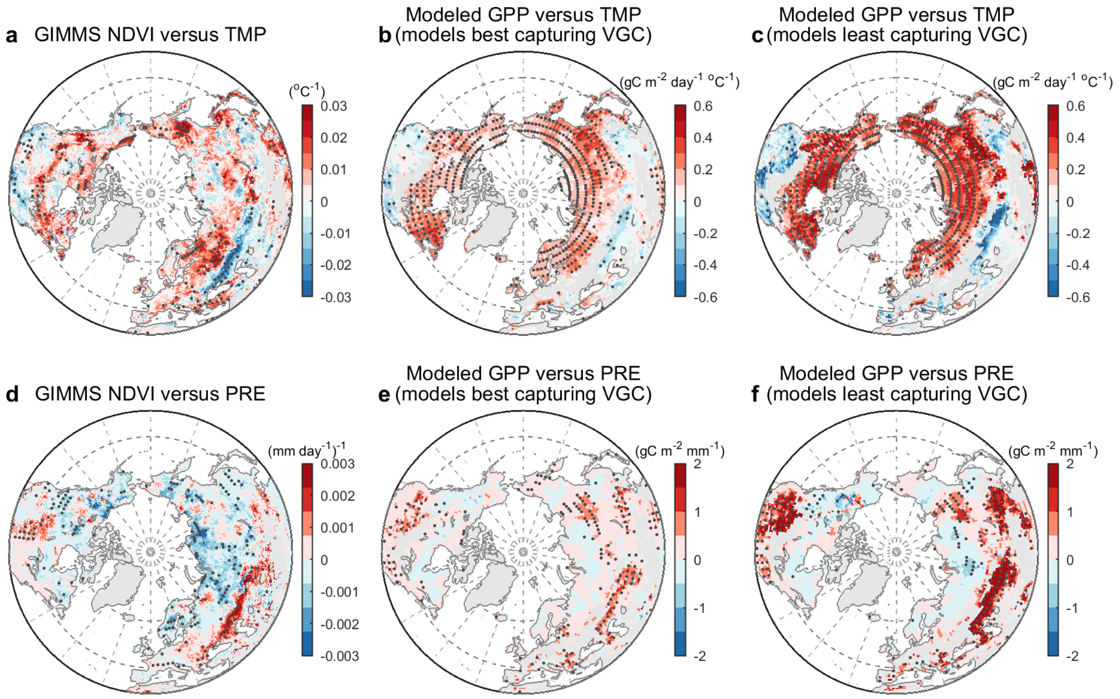

Supplementary Figure 18. Comparison of climatic sensitivities of observed LGS NDVI and modeled LGS GPP. Spatial distribution of the sensitivities of observed LGS productivity (indicated by NDVI) to LGS temperature (**a**) and LGS precipitation (**d**). Panels (**b**) and (**e**) are identical to those of (**a**) and (**d**) respectively, but using estimates from the three models that best identify PGS-to-LGS VGC (JULES, OCN and VISIT, based on Fig. 5i). Modelled GPP is taken as representative of NDVI. Panels (**c**) and (**f**) are identical to those of (**b**) and (**e**) respectively, but for the three models that least capture PGS-to-LGS VGC (LPX-Bern, LPJ-wsl and JSBACH, based on Fig. 5i). In all panels, black dots indicate statistically significant regions at the 95% confidence level.
